# Genetic Resources for Winter Wheat Cultivation Under Severe Cold in Northern Kazakhstan

**DOI:** 10.3390/plants15213258

**Published:** 2026-10-22

**Authors:** Alexey Morgounov, Tatyana Shelaeva, Vladimir Chudinov, Elena Fedorenko, Kadyrzhan Mukin, Kanat Yermekbayev, Beyhan Akin, Vladimir Shamanin, Timur Savin

**Affiliations:** 1 A.I. Barayev Research and Production Center for Grain Farming, Shortandy 021601, Kazakhstan; tatyana.shelaewa@yandex.kz (T.S.); savintimur_83@mail.ru (T.S.); 2 Karabalyk Agricultural Experimental Station, Kostanay 110000, Kazakhstan; ch.den@mail.ru; 3 North Kazakhstan Agricultural Experimental Station, Shagalaly 150311, Kazakhstan; efedorenko2015@mail.ru; 4 Kazakh Research Institute of Agriculture and Plant Growing, Almalybak 040909, Kazakhstan; mukin2010@mail.ru; 5 Institute of Biotechnology and Ecology, Zhetysu University Named After I. Zhansugurov, Taldykorgan 040009, Kazakhstan; kanat.yermekbayev@gmail.com; 6 International Maize and Wheat Improvement Center, Ankara 060031, Türkiye; b.akin@cgiar.org; 7 Department of Agronomy and Plant Breeding, Omsk State Agrarian University Named After Stolypin, Omsk 644008, Russia; vp.shamanin@omgau.org; 8 Research Department, Stev Agro, Astana 010000, Kazakhstan

**Keywords:** winter survival, wheat, grain yield, vernalization, germplasm diversity

## Abstract

Northern Kazakhstan crop production is dominated by spring wheat (12 M ha), with winter wheat occupying a very small area despite its potential for diversification. Winter wheat genetic resources (149 accessions) were evaluated at three sites in Northern Kazakhstan (Kazakh Research and Production Center of Grain, 51.631622 N, 71.031765 E, 2024–26; Karabalyk Agricultural Experimental Station, 53.844492 N, 62.130967 E, 2024–26; North Kazakhstan Agricultural Experimental Station, 54.165411 N, 69.553617 E, 2024 and 2026) and one site in the South (Kazakh Research Institute of Agriculture and Plant Growing, 43.232808 N, 76.703217 E, 2024–26) with the main objective of selecting superior material to be used in production and breeding. Overall, 11 trials were conducted, which included all 149 entries. Variable experimental designs and plot sizes from 1 to 5 m^2^ with 1–2 replications were employed in the study. The evaluation focused on adaptation traits (vernalization requirements in growth chambers, field winter survival, days to heading and plant height), disease resistance, grain productivity and its components. Three study seasons were favorable for winter wheat, with December–February temperatures above the long-term average, reflecting the climate change tendency. Environmental variation across 11 trials allowed detailed evaluation of the germplasm. Winter survival exceeded 60% at six Northern trials out of eight. Overwintering was negatively affected by low temperatures in January and positively by warmer weather in October and November and snowfall in January. Germplasm vernalization requirements remain an important factor in defining winter survival under severe winter stress. Genetic resources developed outside of the region have great potential for Northern Kazakhstan for winter survival, productivity and disease resistance. Germplasm with short and medium plant height and earliness was competitive with the local taller and later-maturing material, thus expanding the diversity and genetic base for breeding. Material that combined positive characters for Northern Kazakhstan included cultivars and breeding lines from Russia (Don and National Grain Centers, Siberian breeding programs) and the USA (Kansas, Montana and Nebraska).

## 1. Introduction

Crop production in Kazakhstan follows a distinct geographic pattern. The South-Eastern and Southern regions have diverse cropping systems (winter cereals, maize, soyabeans, rice and vegetables), whereas the Northern region is dominated by spring wheat [1]. In Northern Kazakhstan, spring wheat is grown on a total area of 12 million ha and accounts for over 98% of the wheat area, with average yield around 1.2–1.7 t/ha depending on the year. Kazakhstan is self-sufficient in wheat grain and exports more than 50% of its stocks, thereby contributing to regional and global food security (https://www.fao.org/faostat/en/#home; accessed on 1 July 2026). According to the Bureau of National Statistics of the Republic of Kazakhstan (https://stat.gov.kz/ru/ accessed on 23 June 2026), in 2025, winter wheat was sown on 10,050 ha in three North Kazakhstan regions (Akmola, Kostanay and North Kazakhstan). By comparison, in the neighboring regions of the Russian Federation (Chelyabinsk, Kurgan, Omsk and Altay), winter wheat occupied more than 200,000 ha (https://eng.rosstat.gov.ru/ accessed on 23 June 2026). Winter wheat cultivation in Siberia has been expanding and is becoming increasingly important for wheat grain production [2]. Across Russia as a whole, the winter wheat area has increased by 9 M ha since 2000 as the crop has moved north and east, replacing spring cereals [3]. The main driver of this shift is that winter wheat generally yields more than spring wheat. Winter wheat also allows producers to use field machinery more efficiently by spreading field work over a longer period. In addition, it increases crop diversification, which helps crop production adapt to climate change. In Northern Kazakhstan, winter wheat still occupies only a negligible area. Winter wheat cultivation is nevertheless associated with certain risks. First, in spring, it is the only crop already established in the field. It is, therefore, exposed to a wide range of diseases (leaf and stem rust, leaf spots and viruses) and pests [4]. These pests include flea beetles, thrips, and stem-boring insects. Over the last two to three years, the wheat curl mite has become a vector of wheat viruses [5]. Second, at least four abiotic stresses can reduce winter wheat yields: autumn drought, low winter temperatures (especially under thin snow cover), alternating thawing and freezing of the soil in spring, and spring drought during crop development [6]. Third, the economics of grain production must be considered. Winter wheat requires early harvest of the preceding crop. If it is sown after fallow, the fallow must be maintained, which adds significant costs per unit area. Profitable winter wheat production depends on combining appropriate crop management with cultivars that are both high-yielding and stable. Modern cultivars that withstand abiotic stresses, pests and diseases, while providing stable high-quality yields, are key to expanding winter wheat production in the region. In Kazakhstan, winter wheat breeding has been carried out at four institutions: the Kazakh Research Institute of Agriculture and Plant Growing (Kaz. RIAPG, Almaty) in the South-East, Krasnovodopad Agricultural Experimental Station (AES) in the South, East Kazakhstan Agricultural Experimental Station in the East, and Karabalyk Agricultural Experimental Station in the North. Only Karabalyk AES is located in the Northern region and develops cultivars adapted to Kostanay, North Kazakhstan and Akmola regions. Overall, 77 winter wheat cultivars are officially registered in Kazakhstan, but only five are approved for cultivation in the North [7]. This includes landmark cultivar Mironovskaya 808, released in 1965.

The main stages of wheat breeding in Kazakhstan are described in *The World Wheat Book* [8,9]. Winter wheat breeding in Kazakhstan began in the 1930s at the Kazakh Research Institute of Agriculture and Plant Growing in the village of Almalybak near Almaty, and a modern breeding program had taken shape by the 1970s. Over the history of the program, more than 30 cultivars have been developed and successfully grown in the Southern and South-Eastern regions. However, despite numerous attempts, none of the cultivars developed at Kaz. RIAPG have proved adapted to the harsh conditions of Northern Kazakhstan. Winter wheat breeding for Northern Kazakhstan and Siberia began in the early 20th century at the West Siberian Breeding Station (Omsk), founded in 1918 [10]. Between 1919 and 1929, more than 2,000 diverse cultivars from different regions were tested there, but none of them overwintered reliably. In five years out of ten, all entries were completely winterkilled. In the 1930s, winter wheat breeding was launched at several sites in Northern Kazakhstan, including Karaganda AES and the Shortandy Experimental Field near Akmola. This work was based on evaluating the extensive Vavilov Institute collection and the use of hybridization. Karaganda AES developed Alabasskaya, which became a benchmark cultivar for winter hardiness. Babaevka, the first winter wheat cultivar from Shortandy, combined high winter hardiness with good yield, earliness and grain quality. Nevertheless, winter wheat breeding at both Shortandy and Karaganda was later discontinued. Winter wheat breeding at Karabalyk Agricultural Experimental Station was initiated in the 1960s and continues to this day. It has produced highly winter-hardy cultivars Komsomolskaya 56 and Komsomolskaya 75, which are adapted to the severe local climate [10]. Since 2010, cultivar Karabalykskaya ozimaya has been approved for cultivation in East Kazakhstan, North Kazakhstan and Kostanay regions [7]. In 2016, Karabalykskaya 101 was entered into the State Register of Breeding Achievements of the Republic of Kazakhstan. Winter bread wheat cultivars Ayaz, Beloglinka, Zere and Gerda, also bred at Karabalyk, are currently in State Variety Trials [11]. Nevertheless, the list of locally bred winter wheat cultivars remains short compared with that of spring wheat. The scientific literature on winter wheat in Northern Kazakhstan is also almost non-existent. The current views on crop prospects in the region are optimistic. They emphasize the need for cultivars that combine strong vernalization requirements, drought tolerance during autumn establishment and responsiveness to favorable conditions in spring and summer [12]. Intensive research is under way to identify promising genetic resources and genetic factors contributing to winter hardiness in Türkiye [13], Iran [14], China [15] and Scandinavia [16]. The physiological mechanisms of winter wheat tolerance to abiotic stresses, including low temperatures, are being studied intensively [15,17,18]. However, the diversity of winter wheat germplasm available for breeding in Northern Kazakhstan is limited, and its adaptation to the conditions of the region remains poorly understood. Since the 2020s, research institutions in Kazakhstan have established collaboration with winter wheat breeding programs in the USA (the universities of Nebraska, Montana, Oklahoma, Kansas and Colorado) and Russia (the National Grain Center in Krasnodar, Don Breeding Center in Rostov and Omsk State Agrarian University), from which modern material, including cultivars and breeding lines, has been obtained. The Genetic Resources Department of Kaz. RIAPG kindly provided winter wheat material received from the International Winter Wheat Improvement Program in Türkiye. In total, more than 260 accessions of the global winter wheat genetic resources were obtained for evaluation under the conditions of Northern Kazakhstan. After initial screening for winter hardiness, disease resistance and agronomic traits, 149 accessions were selected for the current study. The research objectives were the following: (a) define the main weather factors contributing to winter wheat survival in Northern Kazakhstan; (b) estimate the effect of germplasm vernalization requirements, earliness and plant height on winter survival; (c) compare adaptation and agronomic traits in genetic resources of different geographical origin; and (d) select superior winter wheat material for cultivation and utilization in breeding.

## 2. Results

### 2.1. Agronomic Characterization of Experimental Environments

The key winter wheat adaptation trait—winter survival—varied substantially across sites and years (Table 1, Figure S1). As expected, winter damage to plants was minimal at Kaz. RIAPG was exposed to mild winter temperatures in the South. Among the three Northern sites, substantial winter kill was observed at Karabalyk AES in 2025—47.1% average survival for all germplasm tested. The most severe damage was at North-Kazakhstan AES in 2026, where 60% of the germplasm was killed by low temperatures in December amid poor snow cover, with average survival of 16.9% across all accessions. Generally, the 2025 season was the harshest among all, with some damage even at Kaz. RIAPG. The environment at Kaz. RPC Grain provided a more stable evaluation of winter hardiness. From a breeding perspective, these variable years and sites allowed extensive evaluation of the genetic resources for winter hardiness.

The winter wheat growing season in Northern Kazakhstan was 10–30 days later compared with the Southern region. Heading occurred from 3 to 13 June, as compared to mid-May at Kaz. RIAPG. Harvesting in the North took place in the second part of July, allowing at least two months to grow crops or fallow the fields. Plant height in the Northern regions varied within 70–80 cm, unless affected by water stress conditions like those at North Kazakhstan AES in 2026. Combined ANOVA for two 2026 replicated trials (Kaz. RPC Grain and Karabalyk AES) showed high significance for the effects of genotypes, locations (except for grain yield) and their interaction on trait expression (Table S1). Stripe rust occurred annually at the Southern site of Kaz. RIAPG, with severity of 11–47%, while leaf rust dominated in the North, especially at Karabalyk AES. Wheat Streak Mosaic Virus (WSMV) showed a high level of infection at Kaz. RPC Grain in 2026. Winter wheat demonstrated relatively high grain yield at all sites, exceeding 400 g/m^2^ unless affected by winter kill followed by spring drought, which happened at North Kazakhstan AES.

For correlation analysis, the data for all 149 genotypes was used as the statistical unit. Winter survival was positively and significantly associated with grain yield in five trials where these two traits were recorded (Table 2). The magnitude of correlations in the North varied from 0.37 (*p* < 0.001) (Kaz. RPC Grain—2024) to 0.72 (*p* < 0.001) at Karabalyk AES in 2026. This proves that winter hardiness is an important trait contributing to the adaptation of winter wheat to the Northern Kazakhstan environment. Earliness, expressed by the number of days to heading, had a significant negative association with grain yield in three trials out of six. However, the level of correlation was below 0.4, indicating a weak association between the traits. Plant height, in contrast, was positively and significantly associated with grain productivity, with a correlation varying within 0.31–0.45 (*p* < 0.001) in three trials out of seven. Obviously, winter survival was by far more important for winter wheat grain yield among the three traits.

### 2.2. Effect of Weather on Winter Survival Across Sites and Years

**Figure 1:**
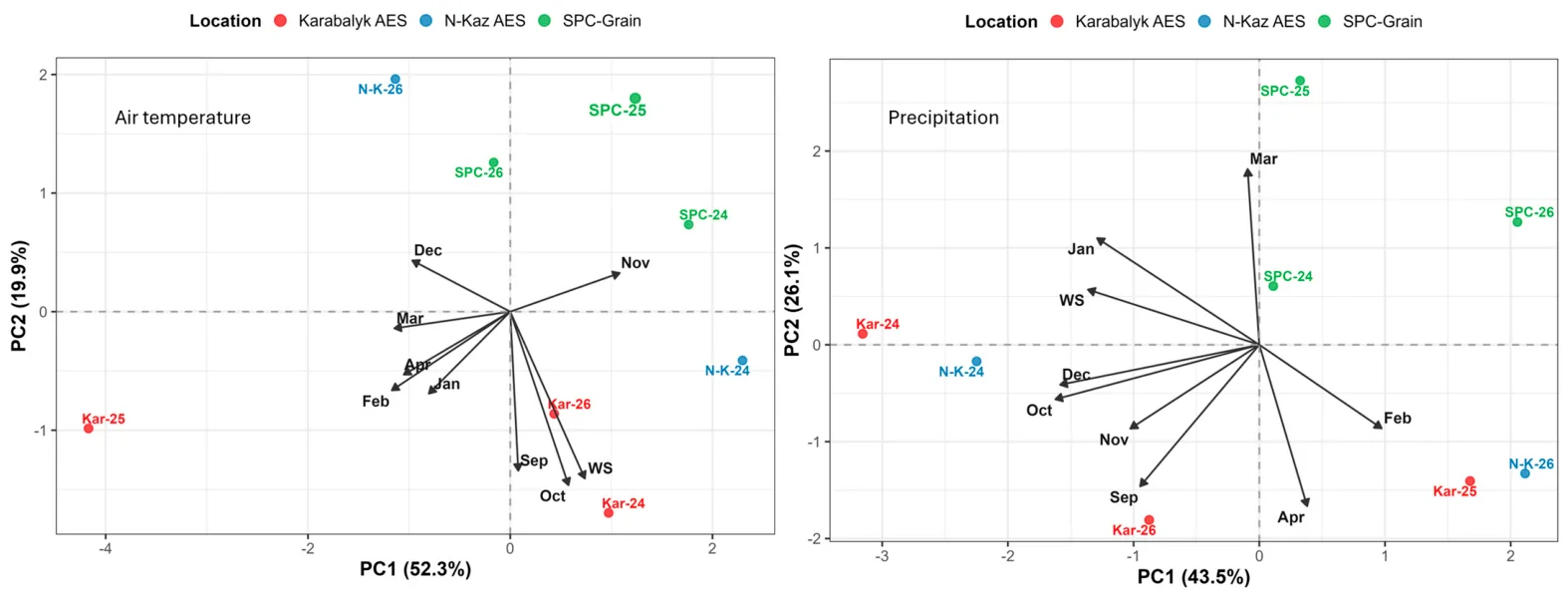
Biplot of monthly air temperature, precipitation and winter survival (WS) at eight Northern winter wheat trials, 2024–26.

Biplot analysis was employed to study the relationship between monthly (September–April) air temperature, precipitation and winter survival at three Northern locations (Figure 1). Two principal components described 72.2% of the variation. The analysis demonstrated a high importance of December in the success of winter wheat. The December temperature vector points in the opposite direction to the winter survival vector, thus showing its negative effect on this trait. Vectors for the fall months of September and October point in a similar direction to winter survival, indicating a positive effect of warmer weather in fall on overwintering. The site and year combinations showed similarity for Kaz. RPC Grain factors in three years, resulting in the formation of a relatively compact cluster. At Karabalyk AES, the combination of factors showed similarity between the 2024 and 2026 seasons.

For precipitation, two principal components described 69.6% of the variation. The vector of snow in January and winter survival almost coincided, showing a strong positive association between these two variables. February precipitation, in contrast, had a strong negative effect on winter survival. The effects of precipitation in other months were not clearly expressed. Again Kaz. RPC Grain trials in three years were relatively close to each other, showing their similarity. Karabalyk AES and North Kazakhstan AES trials in 2024 and in 2025 were closely located, demonstrating their similarity.

### 2.3. Effect of Germplasm Vernalization Requirements, Earliness and Plant Height on Winter Survival

Vernalization requirements were evaluated in growth chambers with exposure of material for 0, 20 and 40 days at +4 °C, followed by placement in the greenhouse. The number of days to heading was recorded for each genotype that reached this stage. The germplasm was grouped into five categories based on vernalization response: (1) Vrn(0)-35–70—no vernalization treatment with material heading withing 35–70 days; (2) Vrn(20)-30–50—20 days of vernalization and heading within 30–50 days; (3) Vrn(20)-51–70—the same but heading within 51–70 days; (4) Vrn(20)-71–100—the same but heading within 71–100 days; and (5) Vrn(40)-35+—40 days of vernalization and heading within 35–45 days plus the germplasm that did not come to heading after 40 days of vernalization. Winter wheat genetic resource evaluation showed the dominance of winter growth habit material, which required 20 days of vernalization [Vrn(20) groups] to initiate development and reach heading. It comprised 132 genotypes (86.3%) (Table 3). Spring growth habit [Vrn(0) group] was demonstrated by only seven entries (4.6%) and true winter types [Vrn(40) groups] by 9.1%. Overwintering for five Vrn groups was compared in three environments: with WS above 80% (Karabalyk AES, 2024 and 2026), 60–80% (Kaz. RPC Grain, 2024–26, North-Kazakhstan AES, 2024) and below 50% (Karabalyk AES, 2024 and North-Kazakhstan AES, 2026) (Table 3). The effect of vernalization duration on winter survival was only significant in the trials with high winter kill. Group Vrn(40)-35+ showed WS of 42.4% in trials with higher winter kill, followed by group Vrn(20)-71–100 at 39.8%. Spring growth habit group Vrn(0)-35–70 and winter groups Vrn(20)-30–50 and 51–70 had significantly lower winter survival by 10–20%. Comparison of the number of days to heading and plant height of five Vrn groups did not show significant differences.

The origin of the germplasm had a substantial effect on vernalization requirements. The material originating from North Kazakhstan and Siberia above 50° N had 42.9% of its genotypes classified in groups Vrn(20)-71–100 and Vrn(40)-35+—most winter-hardy. The share of this material was 13.6% in the germplasm from Turkish programs and 22.2% in the germplasm from the USA Mid-West region (Kansas, Oklahoma, Colorado and Nebraska). These breeding programs are located below 40 °N with occasional winter damage. Material from the southern part of Russia (National Grain Center and Don Center) had 41.3% of its germplasm in two winter-hardy Vrn groups. This is comparable to North Kazakhstan and Siberia germplasm and defines the value of this germplasm for the target breeding region.

Winter hardiness of the four local checks employed in the study also proved the importance of vernalization requirements. Average winter survival of the four checks across all Northern trials was: Vavilov, Vrn(0)-35–70 group—52.5%; Steklovidnaya-24, Vrn(20)-51–70 group—51.4%; Skipetr, Vrn(20)-51–70—72.5% and Gerda, Vrn(20)-71–100—75.2%. However, at North-Kazakhstan AES, winter survival was, respectively, 0, 10, 60 and 80%. This connects vernalization requirements with WS under severe winter conditions. It also provides confidence in using these four genotypes as local checks in the current study and future studies.

**Figure 2:**
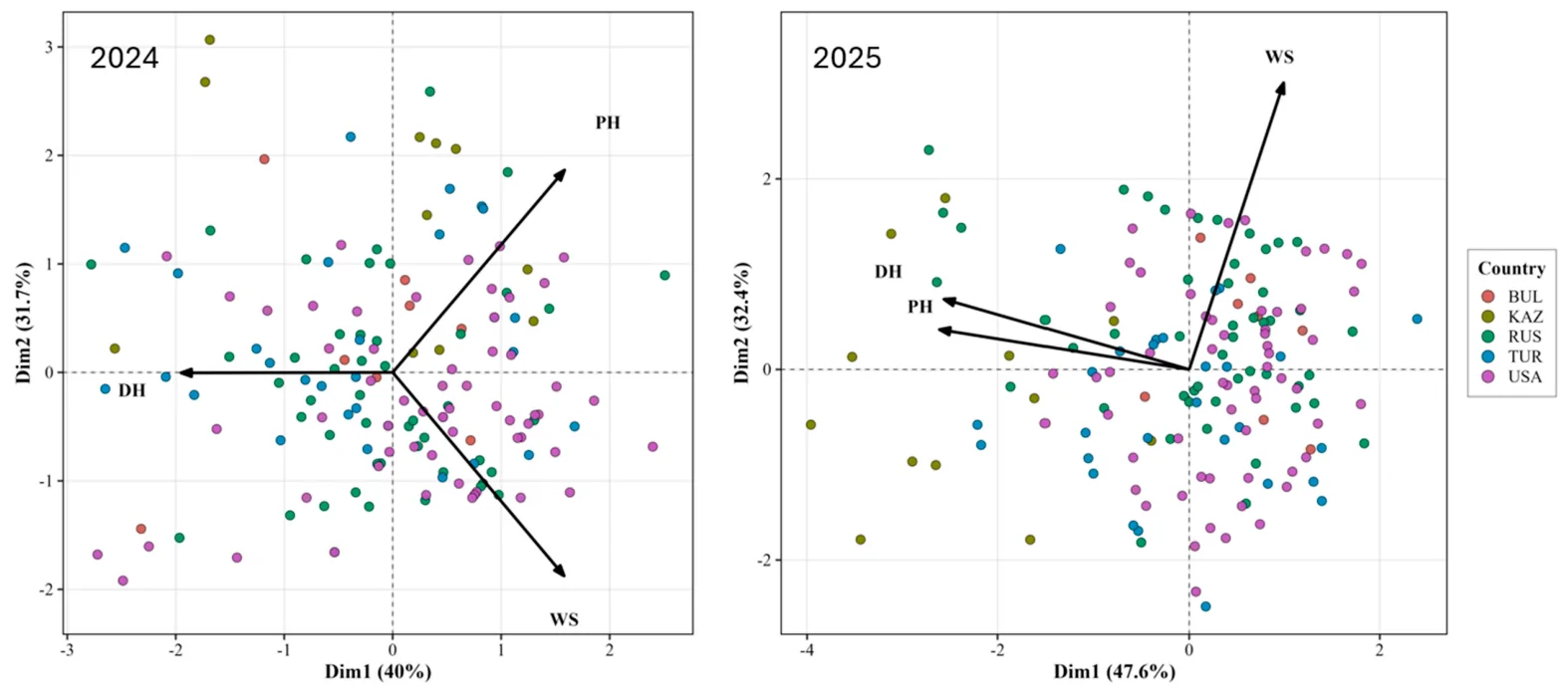
Biplot of winter survival, number of days to heading and plant height at Kazakh RPC Grain in 2024 and 2025 (DH–dys to heading, PH–plant height).

The relationship between winter survival and the key adaptation traits like earliness and plant height across all genotypes is reflected in the biplots presented in Figure 2 and Figure S2 for Kaz. RPC Grain. The relationship between the three traits in 2024 at Kaz. RPC Grain was quite independent, with three respective vectors pointing in different directions without much relationship among them (Figure 2). In 2025 and 2026, the number of days to heading and plant height were positively associated, as their respective vectors were close to each other. These two traits were also positioned at around 90° to the WS vector, indicating that no clear association was apparent in the PCA biplots. However, some significant correlations between WS and days to heading calculated for all genotypes were observed at Karabalyk in 2024 (−0.27, *p* < 0.001), North Kazakhstan AES in 2024 (−0.56, *p* < 0.001) and in 2026 (0.36, *p* < 0.001) (Table S2). It appears that earliness was significantly associated with WS either positively or negatively depending on the environment. Plant height had a significant positive correlation with WS at Karabalyk AES in 2024 (0.18, *p* < 0.05) and in 2026 (0.26, *p* < 0.01) and at North Kazakhstan AES in 2026 (0.43, *p* < 0.001). Plant height contribution to WS was highest in the trial with the highest winter kill—North Kazakhstan AES in 2026.

### 2.4. Adaptation and Agronomic Traits in Genetic Resources of Different Origins

In order to evaluate the effect of germplasm origin on its performance, the genetic resources were combined into six groups: (1) North Kazakhstan (Karabalyk AES) and Siberia (Omsk SAU and other programs); (2) South Russia (Don and National Grain Centers); (3) Bulgaria (various programs); (4) Türkiye (all programs); (5) USA Mid-West (Colorado, Kansas, Nebraska and Oklahoma) and (6) USA North-West (Montana and Washington). Average winter survival across trials was significantly higher in the germplasm originating from Kazakhstan and Siberian breeding programs—70.4%, South Russia (69.1%) and the two US groups (Table 4). Türkiye material had the significantly lowest WS (60.2%). However, when the germplasm was exposed to severe winter kill at North-Kazakhstan AES in 2026, the highest survival was demonstrated by group 1—46.5%, significantly higher compared to South Russia (18.8%) and the North-West USA group (18.4%). Turkish and Bulgarian germplasm were almost entirely killed, while US Mid-West material had WS below 10% on average. These results demonstrate that local North Kazakhstan and Siberian germplasm cannot be underestimated for cultivation under extreme winter conditions.

For earliness expressed by the number of days to heading, the differences between the germplasm groups were within 2–3 days. The germplasm samples from Kazakhstan, Siberia and Montana SU were significantly later by 2–3 days (Table 4). Kazakhstan and Siberian material represented traditional tall varieties with an average plant height of 98.2 cm, significantly taller compared to all other groups, while material from South Russia was the shortest (76.5 cm). Winter wheat from the USA maintained a height within 80–83 cm.

Stripe rust in Kazakhstan is confined to winter wheat in the Southern part of the country, while leaf rust occurs across all regions. The highest level of resistance to stripe rust was demonstrated by the germplasm from Montana and the state of Washington (US North-West group), with an average severity of 20.4%. The second most resistant group was from South Russia—35.2%—while infections in all other groups exceeded 45%, with the highest susceptibility in the Kazakhstan and Siberian group (65.5%). Bulgarian material showed the highest resistance to leaf rust, with a severity of 26.9%, while in all other groups, it exceeded 40%, again with high-latitude germplasm (North Kazakhstan and Siberia) being most susceptible (64.8%). Symptoms of WSMV were observed once at Kaz. RPC Grain, with a negative effect on productivity (correlation between WSMV score and grain yield = −0.76, *p* < 0.001) (Figure S3). The lowest virus severity readings were in USA North-West germplasm (20.3%), followed by the Kazakhstan–Siberia and South Russia groups (23.3 and 24.5%, respectively).

Analysis of spike productivity indicated that material from Kazakhstan and Siberian programs had long spikes (9.7 cm), a high number of grains per spike (>45) and average grain size with a TKW of 38 g. The Russian germplasm from Don and National Grain Centers had smaller spikes (8.8 cm), 43 grains per spike and a TKW of around 39 g. The USA material had smaller spikes (8.0–8.4 cm), a variable number of grains per spike (40–44) and small grains with a TKW of 36 g for the Mid-West group and 33 g for North-West genotypes. The germplasm from Bulgaria and Türkiye was characterized by an average spike size (8–10 cm), a high number of grains per spike and 1000-kernel weight exceeding 40 g. Thus, the genetic resources with different geographical origins showed complementary adaptation and productivity traits, proving their value for breeding winter wheat in Northern Kazakhstan conditions.

**Figure 3:**
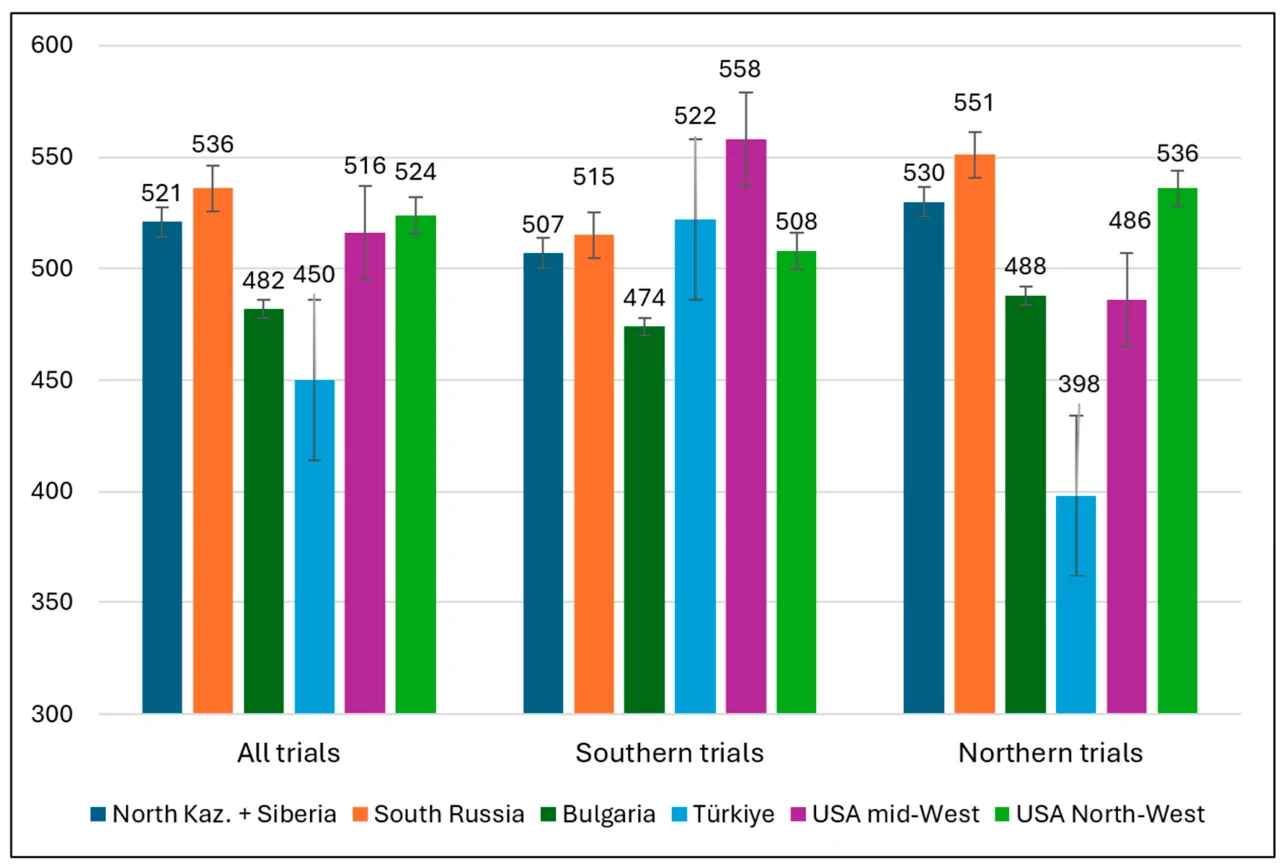
Grain productivity (g/m^2^) of winter wheat germplasm of different origins, 2024–26.

Winter wheat demonstrated relatively high grain productivity across the testing sites and years above 450 g/m^2^ (Figure 3, Table S3). Four groups showed significantly higher grain yield: North Kazakhstan and Siberia, South Russia and the two US groups. Türkiye material was the lowest yielding. In two Southern trials (Kaz. RIAPG, 2025 and 2026), USA Mid-West germplasm significantly outyielded (558 g/m^2^) all other groups except the Turkish group (522 g/m^2^). At Northern sites (without the severely damaged North-Kazakhstan AES trial in 2026), South Russia germplasm was the highest yielding (551 g/m^2^), followed by the USA North-West group (536 g/m^2^) and Kazakhstan–Siberia material (530 g/m^2^). A significance test placed USA Mid-West and Türkiye material below the three highest yielding groups in the Northern trials. While this analysis broadly described the advantages and disadvantages of genetic resources from different origins, there is substantial variation for target traits within each group.

### 2.5. Superior Winter Wheat Genetic Resources for Utilization in Northern Kazakhstan

The stability analysis was performed for winter survival (nine trials) and grain productivity (six trials), including all trials. The mean value of the trait was the first criterion for selecting promising genotypes (Table 5). The Lin and Binns [19] superiority measure (Pi) was the main stability criterion as it combines high performance and consistency in one value. A low Pi means the genotype stayed close to the best entry in each environment. Eberhart and Russell [20] parameters (Bi and R^2^) were used to describe how genotypes respond to the environment. CV% served as a simple descriptive measure of variation across environments. Table 5 presents the top ten performers for each trait, and Table S4 presents the 30 best genotypes. The data for the best local check is also included. The breeding line from Russia (BC2F2 U346(113/96)/D//D/3/D) showed the highest winter survival (89.5%), though it was not significantly different compared to the best local check—Gerda (75.1%). However, it had the lowest Pi, indicating that it is close to the best-performing genotypes in each trial. It showed low response to environment (Bi = 0.13), and the linear regression line poorly explained its performance (R^2^ = 0.09). The performance of the second-best Siberian line, KP-134-18, was very close to that of BC2F2 U346(113/96)/D//D/3/D. Cultivar Stanichnaya from the National Grain Center (Russia) combines high winter survival (81.2%), low CV and Pi, and a below-average response to environment (Bi = 0.66), which is well explained by the linear regression (R^2^ = 0.91). The top ten group includes four more genotypes from South Russia (National Grain and Don Centers) and three from the USA (two from KSU and one from UNL). They all vary in winter survival stability but certainly represent valuable genetic resources for winter wheat production and breeding in Northern Kazakhstan.

Surprisingly, local Kazakh germplasm from Karabalyk AES did not perform well enough to enter the top 10 list for winter survival, and there were only four entries in the top 30 list. However, local germplasm performed best at North Kazakhstan AES in 2026 with severe winter kill (Table S5). With an average trial WS of 17%, seven entries from Karabalyk AES demonstrated winter hardiness of 60% and higher. Cultivar Gerda and line Lutescens 748-95 showed grain yield above 100 g/m^2^. Five genotypes from South Russia entered the top 20 list at this location, but their grain yield was very low. Two USA genotypes (MT1745 from MSU and Guardian from CSU) survived winter relatively well (WS > 60%) but barely produced any yield. Once again, the value of local material for environments with severe winters is certainly high.

For grain yield stability across all sites and years (six trials), USA germplasm dominated, contributing six genotypes to the top 10 list (Table 5) and 15 to the top 30 list (Table S4). The highest yielding group includes KS14DH0024-28, USA-KSU (560 g/m^2^), Zhiva, RUS, National Grain Center (547 g/m^2^), Lutescens 748-95, KAZ-Karabalyk AES (535 g/m^2^), Snowmass 2.0, USA-CSU (529 g/m^2^), and NE17544-2, USA-UNL (513 g/m^2^). Their grain yield CV values exceeded 45%, and they were all responsive to environmental conditions with Bi above 1. Interestingly, the top 10 lists for WS and grain yield do not overlap. This indicates that in a broad winter wheat environment, including Southern locations, high WS is not necessarily a major factor defining productivity.

The results of two replicated Northern trials (Kaz. RPC Grain and Karabalyk AES) for WS and grain yield are summarized in Table S6. The breeding line from KSU—KS13DH0030-29—significantly outyielded the highest yielding local check—Gerda: 702 vs. 571 g/m^2^. High-yielding germplasm (>600 g/m^2^) also included USA material (Robidoux, Guardian, Judee, MTFH20170), South Russia material (Svarog, L.6471 k 13, Konkurent, Donna) and one local breeding line, Lutescens SP-1-3269.

A large number of stripe and leaf rust-resistant genetic resources were identified during the study. Table S7 provides a list of 45 entries resistant to stripe rust, with infection severity below 20%, while the susceptible check infection was 100%. Likewise, 40 genotypes resistant to leaf rust with severity below 25% are included in Table S7. Germplasm resistant to WSMV with infection severity below 10% comprises 15 genotypes, primarily from the USA (Table S8). Overall, the superior genetic resources identified in this study represent a wealth of diversity suitable for potential cultivation in Northern Kazakhstan and utilization in the breeding programs.

## 3. Discussion

### 3.1. Study Limitations

Unbalanced experimental designs, including unreplicated trials and an unbalanced data set, represented a challenge for the analysis. Spatial adjustment of the traits in unreplicated trials provided a more confident evaluation of the germplasm. The main traits used in the study (WS, days to heading, plant height, disease resistance) have relatively high inheritance, and uniform unreplicated trials contributed to their evaluation. Overall, this multilocational framework allowed implementation of the main study objective—comprehensive evaluation of winter wheat genetic resources for Northern Kazakhstan and selection of promising germplasm for utilization in the region. The material used in the study went through preliminary screening to eliminate germplasm with poor winter hardiness and leaf rust susceptibility. This affected the diversity of the study set. However, from a practical perspective, selected genetic resources represent more value. The presence of the checks with variable winter hardiness allowed benchmarks for evaluation. The study germplasm did not include the genetic resources from similar ecologies like Canada, Scandinavia, the USA (North Dakota), Central Russia, the Volga region, and possibly others. Exchange of germplasm with these regions, testing them and selecting prospective genotypes is highly valuable. Unfortunately, stricter rules apply to seed exchange for both breeder’s rights protection and to meet phytosanitary regulations [21].

The study did not cover physiological and genetic factors contributing to winter hardiness and its components like frost tolerance, tolerance to late frost in spring, resistance to snow mold, hardening capacity and others. Some traits (coleoptile length) are being studied, while others are planned to be used on a smaller set of germplasm. A genomic study is underway to test the effects of known molecular markers on trait expression, as well as to conduct Genome-Wide Association Mapping using the phenotypic data from the current study. The important topic of grain quality was also beyond the current study.

### 3.2. Winter Wheat for Adaptation to the Conditions of Northern Kazakhstan

Three study seasons (2024–26) coincided with less severe winters, with monthly air temperatures in December and January 1–3 °C above the long-term average. Amid this weather, winter wheat survival at six Northern trials out of eight exceeded 60% and was suitable for successful crops. Only one trial was severely affected by frost, with winter kill exceeding 80%. However, even in this trial, some material demonstrated suitable winter survival and produced grain. The results indicated a negative effect of air temperature in December and a positive effect of September and October temperature on winter survival. Snow in January also had a positive effect on winter survival. Grain yields of winter wheat at Kaz. RPC Grain and Karabalyk AES were 40–70% higher compared to spring wheat breeding trials in the same season. A yield comparison of the two crops over 22 years (1995–2016) at Karabalyk AES demonstrated an advantage of spring wheat—3.4 t/ha vs. 2.4 t/ha [11]. However, the recent warming and favorable weather benefitted winter wheat more than spring wheat, resulting in much higher yields of this crop. Overall, the trial results cautiously suggest suitability of winter wheat for Northern Kazakhstan conditions, as well as the need for selection of relevant cultivars.

The study presented the results of a comprehensive assessment of a large international winter wheat collection under the conditions of Northern Kazakhstan, the first since the 1990s. Two institutions involved in the study (Kaz. RPC Grain and North-Kazakhstan AES) planted winter wheat evaluation nurseries for the first time in 20–30 years. The main outcomes are the following. First, high winter hardiness in winters with low temperatures was associated with plant height and better overwintering of medium and tall varieties [22]. An example is the well-known variety Mironovskaya 808, which is still released for the North of Kazakhstan. This study confirmed that the varieties developed at Karabalyk AES and in the adjacent regions of Russia (Kurgan and Omsk) exceeded 100 cm in height and are prone to lodging under favorable conditions [23]. Nevertheless, medium- and short-stature varieties from Russia and the United States were not inferior to local varieties and checks in winter hardiness, and some even surpassed them. However, in the trial with high winter kill (North-Kazakhstan AES, 2026), local Kazakh material demonstrated its superiority for winter hardiness, though few introduced genotypes were competitive and could be used in breeding. Producers prefer short-stature varieties of spring and winter wheat due to their technological advantage [24]. The role of plant height in ensuring high winter hardiness deserves a more detailed study in various cultivation conditions of Northern Kazakhstan.

Secondly, high winter hardiness is associated with the vernalization period [25]. In this study, the requirements for vernalization were documented for the material. Winter wheat that was exposed to 20 days and 40 days of vernalization and headed within 71–100 and 35–45 days, respectively, showed higher winter survival compared to spring growth habit or winter wheat with lower vernalization needs under more severe winter conditions. Identification of molecular markers associated with Vrn genes is underway and will contribute to a better understanding of the role of vernalization in winter hardiness.

The only winter wheat breeding program for Northern Kazakhstan is located at Karabalyk AES, where traditional breeding methods have been utilized and commercial cultivars developed [11]. Nevertheless, the breeding material is characterized by a relatively high degree of homogeneity, as evidenced by this study and visual assessments during the study. The local germplasm mainly belongs to the botanical variety of lutescens, medium- and tall-stature, relatively late-maturing, and susceptible to rusts. The superior genetic resources selected in this study will expand phenotypic variability and introduce new genes that contribute to the adaptation of this crop to the conditions of the region.

### 3.3. Prospects for the Use of Winter Wheat Genetic Resources in Kazakhstan

Evaluation of extensive and genetically diverse germplasm identified valuable material with high winter hardiness; optimal plant height; resistance to stripe, leaf rust, and WSMV; and high productivity in Northern and Southern regions of Kazakhstan. A similar assessment of the winter wheat collection was carried out in the Omsk region, and some accessions selected in Kazakhstan also showed their potential in Western Siberia [26]. The best genetic resources identified for winter survival and grain yield possess broad adaptation and represent high genetic diversity. Cultivar Stanichnaya (Don Center) demonstrated high winter hardiness in neighboring regions of Russia [27]. Variety Zhiva from Krasnodar has an effective root system, contributing to high yield performance [28]. Cultivar Panhandle showed a high level of winter hardiness in Western Nebraska [29]. The breeding line with the highest winter hardiness, BC2F2 U346(113/96)/D//D/3/D, originated from wide crosses with *Aegilops* sp. [30].

Material from Siberian breeding programs and Kazakh germplasm were similar in adaptation and require shortening of plant height and introduction of leaf rust resistance. The germplasm from Russia Don and National Grain Centers has values for winter hardiness and yield potential. US material represents a good source for earliness, yield stability and disease resistance. The selected material can be used as parental forms in crosses. Some material can be considered as potential variety candidates, subject to restrictions of originating breeding institutions. The study also emphasizes the importance of germplasm exchange in improving adaptation to severe winter environments and achieving genetic gains for productivity and other traits.

Promising genetic resources of winter wheat in breeding can be much more effective using modern tools and technologies. The study demonstrated the value of multilocational evaluation and complementarity of three Northern sites supported by the established program in the South-East. This network may need optimization and formal agreement to operate effectively. Studies conducted in Kazakhstan have identified molecular markers of winter wheat associated with productivity and its components [31], including resistance to yellow rust and smut [32]. Interesting results on genomic markers of winter hardiness were obtained in Lithuania and Russia [16,33]. Screening for and validating the known markers and selecting and using the most promising for the conditions of Kazakhstan will significantly increase the efficiency of selection for complex traits such as winter hardiness.

Accelerated breeding makes it possible to develop new wheat cultivars much faster by obtaining several generations per year. A modern platform for accelerated breeding and phenotyping has been established and effectively operates at Zhetysu University, named after I. Zhansugurov [34]. It allows obtaining at least two generations of winter wheat per year, as well as evaluating the material for important traits like vernalization requirements. The cooperation of breeding programs with the university will accelerate and increase the efficiency of genetic resource utilization.

## 4. Materials and Methods

### 4.1. Winter Wheat Material

The collection of genetic resources included in the study comprised 149 accessions from 5 countries (Table S9). It was selected from a larger set of 268 accessions based on a preliminary evaluation in 2022–23 at Karabalyk AES and Kaz. RIAPG. The germplasm with poor winter survival and leaf rust susceptibility was discarded from further testing while maintaining the geographic representation. The discarded material primarily originated from Türkiye (50%), the USA (15%), Russia (11%) and Kazakhstan (8%). The selected research germplasm originated from more than 15 breeding programs and included released cultivars, new cultivars and breeding lines. The main contributors to the collection were: the USA (58 entries, including 18 from Kansas State University, 13 from Oklahoma State University, 9 from University of Nebraska—Lincoln and 8 from Montana State University), Russia (46 entries, including 18 from the National Grain Center at Krasnodar, 11 from Don Breeding Center at Rostov and 17 from various breeding programs in Siberia), Türkiye (28 entries, including 10 from Bahri Dagdas International Agricultural Research Center in Konya and 8 from Trakya Agricultural Research Institute in Edirne), and Bulgaria (8 entries). The seeds of most of the material were obtained directly from the breeding programs or through the International Winter Wheat Improvement Program (Türkiye-CIMMYT-ICARDA) based in Ankara, Türkiye. Kazakhstan germplasm was represented by 9 cultivars and breeding lines from Karabalyk AES, the only winter wheat breeding program in Northern Kazakhstan.

The main continuous local check was cultivar Steklovidnaya-24 (widely adapted variety from Kaz RIAPG in South-Eastern Kazakhstan with average winter hardiness). This local check was included in all unreplicated trials every 25 to 50 entries, allowing statistical adjustment of the trait values. Additional local checks were the varieties Vavilov (new facultative wheat cultivar from Kaz RIAPG with poor winter hardiness), Skipetr (Russian widely adapted cultivar also grown in Northern Kazakhstan), and Gerda (new winter-hardy cultivar from Karabalyk AES). These checks were included as a single plot in each trial, with the exception of Vavilov, which was repeated every 30 entries in all 2024 trials. The main objective of the additional checks was to provide varying degrees of winter hardiness and, in the case of Skipetr, a comparison to winter wheat commercially grown in Northern Kazakhstan.

### 4.2. Experimental Sites and Field Trials

Phenotyping and agronomic evaluation of the collection was carried out over three winter wheat seasons (2023–24, 2024–25 and 2025–26, referred to in the text by the harvest year—2024, 2025 and 2026, respectively) at three research institutions in Northern Kazakhstan (Kazakh Research and Production Center for Grain Farming, named after A.I. Barayev, Kaz. RPC Grain, Karabalyk AES and North Kazakhstan AES) and in the South-East Almaty region at the Kazakh Research Institute of Agriculture and Plant Growing (Kaz. RIAPG) (Table 6). All Northern sites are located above 50° North with severe winters and average January temperatures of around −16 °C. The summer season is short, from May to September, with yearly precipitation in a range of 300–450 mm. In contrast, Kaz RIAPG conditions are characterized by mild winters, hot summers and high rainfall exceeding 650 mm.

The soil at the experimental sites was represented by two types of chernozems: ordinary (Karabalyk and North Kazakhstan AES) and southern (Kaz. RPC Grain). No soil analysis at the trials plots was conducted, but generally, these soils are characterized by relatively high organic matter content (3–5%), potassium availability, and variable nitrogen and phosphorus requirements depending on the preceding crop and other factors. In the South-East at Kaz. RIAPG, soil was light chestnut with an organic matter content of 2–3% and average availability of potassium, nitrogen and phosphorus. All trials were conducted under rainfed conditions. The agronomic practices used in the trials reflected general regional practice for winter wheat: sowing in the first half of September in the North and in the second part of October in the South. The preceding field was fallow, with application of nitrogen fertilizer at a rate of N30 in spring and chemical weed control with herbicides. Fungicide protection was not used.

### 4.3. Field Trial Description

Experiments were laid out at research fields, with plot sizes of around 1 m^2^ in 2024, around 3 m^2^ in 2025 without replications and 4–5 m^2^ in 2026 with one or two replications (Table 7 and Table S10). Insufficient seed amount was the main limiting factor for using small unreplicated plots in the first and second years. Overall, 11 experiments were conducted, including 9 unreplicated and 2 replicated. Unreplicated trials followed a systematic germplasm layout and repeated check/s to account for spatial variation. Two replicated trials followed an RCBD design. Details on experimental units, plot dimensions and area, borders and local check frequency are presented in Table S10.

Evaluation was carried out for winter survival (WS) in April, after plants started spring vegetation and there was clear differentiation between the green surviving plants and brown plants killed during winter. Visual evaluation was conducted by the same individual across all trials. The percentage of green plants that survived in spring out of all green and brown plants on the whole plot was recorded as winter survival. This method was used at Karabalyk and North-Kazakhstan AES and Kaz. RIAPG. A different method was used at Kaz RPC Grain: the number of plants after emergence in fall and green plants after the start of spring vegetation were counted in fixed sampling areas (0.25 m^2^) within plots excluding border rows. Winter survival was calculated as the percentage of green plants in spring out of all plants in autumn in the same fixed sampling area. Field evaluation included number of days to heading (DH, presented as number of days from 1 May), plant height and disease resistance under natural infection. Spike productivity traits included spike length, number of spikelets per spike, number of grains per spike and spikelet, and 1000-kernel weight (TKW) using a sample of five random spikes collected from the plot prior to harvest. The assessment followed the methodology presented in the CIMMYT physiological breeding manual [35]. For grain yield evaluation, the whole plot was harvested, threshed, dried, and weighed, and productivity was calculated in grams per 1 m^2^.

The list of traits evaluated in the trials varied depending on the site and year (Table 7). Winter survival was the only character observed across all locations and years. In 2024, field data on the number of days to heading, plant height and spike productivity was obtained at three locations, namely, Kaz. RPC Grain, Kaz. RIAPG and Karabalyk AES, while the grain yield was obtained from Kaz. RPC Grain. In 2025, the trial at North Kazakhstan failed due to late sowing, and no data was obtained. Two other sites provided only data on winter survival. Grain yield was not recorded at Kazakh Grain RPC due to bird damage or at Karabalyk due to early drought. In 2026, the most complete data was obtained across the majority of the traits, including grain yield. All traits recorded and presented in Table 7 were used for analysis.

For diseases, observations were made for stripe rust at Kaz. RIAPG in all three seasons and leaf rust at Kaz. RIAPG (2024 and 2026), Karabalyk and North Kazakhstan AES (2024). Wheat Streak Mosaic Virus (WSMV) affected winter wheat at Kaz. RPC Grain in 2026. Other diseases like powdery mildew and Septoria were observed in the trials with low infection and recorded but not used in the analysis. For rusts, disease severity on flag and second leaf (%) was recorded following the CIMMYT Manual [36]. For WSMV, percentage of virus spread from bottom to top was recorded.

Unbalanced experimental designs, including unreplicated trials and unbalanced data sets, represented certain challenges for the analysis. These are addressed in the Study Limitations Section in the Discussion Section. However, adjustment of the traits in unreplicated trials to account for spatial variation was performed using check-plot correction.

### 4.4. Vernalization Tests

The germplasm panel was grown in 50-well trays in two replications using a Randomized Complete Block Design (RCBD) in which trays were treated as blocks and each tray was independently randomized. Two seeds of each accession were planted in each well filled with a growing substrate containing 65% peatmoss, 18% perlite, 12% sand and 7% biohumus. The trays were placed into a Controlled Environment Chamber (CEC, Being Technology Co., Ltd., Beijing, China) and vernalized at +4 °C under 8 h of light and 16 h of darkness at 50% humidity for 20 and 40 days. Upon completion of the vernalization period, the plants were transferred to a speed breeding platform, where they were grown at 20–22 °C under 22 h of light and 2 h of darkness until the end of their growth cycle, as described by Yermekbayev et al. [34]. Light intensities were maintained in the range of 500–700, depending on the developmental stage. The trays were periodically (once a week) moved in both vernalization and the greenhouse to avoid exposure to uneven conditions. The number of days from placement in the greenhouse to heading was recorded for each genotype that reached this stage.

Three vernalization treatments were applied: Vrn(0)—control without exposure to vernalization; Vrn(20)—20 days of exposure to vernalization and Vrn(40)—40 days of vernalization. The germplasm was grouped into five categories based on vernalization response: (1) Vrn(0)-35–70—no vernalization treatment with material heading withing 35–70 days; (2) Vrn(20)-30–50—20 days of vernalization and heading within 30–50 days; (3) Vrn(20)-51–70—the same but heading within 51–70 days; (4) Vrn(20)-71–100—the same but heading within 71–100 days; and (5) Vrn(40)-35+—40 days of vernalization and heading within 35–45 days plus the germplasm that did not come to heading after 40 days of vernalization. Group 1 represents spring growth habit material; groups 2–4 represent winter growth habit with increasing vernalization requirements, while group 5 is likely to be true winter growth habit types. The five categories were established without a relationship to winter survival and showed a normal distribution pattern. This methodology is similar to the approach used by Penrose et al. [36].

### 4.5. Weather Conditions

Three experimental seasons were generally favorable for winter wheat production, with winter month temperatures and precipitation above the long-term average (Table 8 and Table S11). The coldest month, January, had higher average air temperatures during the last three years—13.6–16.2 or 1–3 °C—compared to the long-term observations. Weather in December was also higher by 0.7–4.2 °C across three Northern locations. Critical spring months also demonstrated higher temperatures without the return of frost after the start of vegetation. June was slightly warmer compared to the long-term average across all sites. Considering the seasonal air temperature average, Kaz. RPC Grain demonstrated the highest increase of 2.8 °C, followed by North-Kazakhstan AES (+1.6 °C) and Karabalyk AES (+1.1 °C). Temperatures above the long-term average were also recorded at the Southern location of Kaz. RIAPG.

Seasonal precipitation in 2023–26 at Kaz RPC Grain was close to the long-term average, with a drier April but a wetter May. At Karabalyk AES, December, May and June experienced higher precipitation, and seasonal figures were 40% higher compared to the long-term average. May and June were also wetter at North-Kazakhstan AES. The season of 2024 had higher precipitation compared to other seasons across all sites. Overall, weather conditions were sufficiently variable across sites and years, allowing a comprehensive evaluation of winter wheat germplasm for adaptation and productivity traits.

### 4.6. Statistical Analysis

All experimental data were integrated in Excel. Basic descriptive statistics were also performed in Excel: average values, standard deviations, standard errors of the mean, and correlation coefficients. More advanced statistical analysis was performed in R software version R 4.5.3. Adjustment of the traits in unreplicated trials to account for spatial variation was performed using check-plot correction following Chandra [37] and the augmented design moving means method (https://ecommons.cornell.edu/entities/publication/22e171a6-71d7-422a-9de5-9d7cc372ccd0, accessed on 8 September 2026), implemented via inverse-distance weighting, with adjustment applied selectively per trait based on leave-one-out cross-validation of the check plots. Adjustments were made in six out of nine unreplicated trials for overall 20 traits (Table S12). Thus, we used adjusted values for analysis.

Combined analysis of variance for agronomic traits across two replicated 2026 trials (Kaz. RPC Grain and Karabalyk AES) was performed using an RCBD, with location, genotype, and their interaction as fixed effects and replication nested within location as a random effect.

To characterize and relate weather parameters to observed winter survival, monthly mean air temperature and monthly total precipitation (September–April) at each site–year were each subjected to a principal component analysis (PCA), together with the site–year’s mean winter survival values. Variables were standardized to unit variance prior to analysis (i.e., PCA computed on the correlation matrix). Biplots display site–year scores in the plane of the first two principal components, colored by location, with the monthly climate variables and winter survival represented as vectors from the origin; the percentage of total variance explained by each axis is reported in the axis label. A similar approach was used for PCA involving WS, days to heading and plant height.

To determine the significance of differences in average winter hardiness between five vernalization requirements groups we used one-way ANOVA, with Vrn group as the sole factor. Homogeneity of variance was confirmed by Levene’s test prior to analysis. Where the ANOVA F-test was significant, pairwise differences among group means were tested using Tukey’s honestly significant difference (HSD) test (α = 0.05); means not sharing a common letter differed significantly. Group sample sizes were unbalanced (*n* = 7–83 lines per group), which Tukey’s test accommodates directly. The same approach was applied while comparing mean values of germplasm groups with different origins for agronomic traits.

Stability analysis for WS and grain yield was conducted using multilocational data. For each trait, entry performance across site–years was summarized by the mean and coefficient of variation (CV%). Stability performance was assessed using two complementary statistics. Firstly, the Lin and Binns superiority measure [19], which quantifies each entry’s mean squared deviation from the best-performing entry observed in each environment (lower values indicating consistently near-top performance across environments). Secondly, Eberhart and Russell [20] joint regression of each entry’s environmental values on the environmental index (site–year mean minus grand mean), yielding a regression slope (bi, where bi ≈ 1 indicates average environmental responsiveness, bi < 1 indicates below-average responsiveness and greater buffering, and bi > 1 indicates above-average responsiveness with greater exposure to unfavorable environments) and its coefficient of determination (R^2^), reflecting how well each entry’s response conforms to the fitted regression line.

## 5. Conclusions

1. Winter wheat in Northern Kazakhstan trials proved likely suitable for local conditions with relatively stable winter survival and high grain yield. Winter survival is negatively affected by low temperatures in January and positively by higher temperatures in October and November and snowfall in January. The risk of substantial winter kill remains but was only observed in one trial out of eight. Under severe weather conditions, some genotypes showed high winter survival and produced grain yield. Vernalization requirements remain an important factor in defining winter survival, especially under severe winter stress. Selection of well-adapted, winter-hardy, productive and disease-resistant cultivars is an important factor in the success of this crop. Production technology, though not addressed in the study, remains another important factor for crop cultivation.
2. The evaluation results of diverse winter wheat genetic resources proved that material developed outside of the region in milder climates has great potential in Northern Kazakhstan for winter survival, productivity and disease resistance (leaf rust and WSMV). Germplasm with short and medium plant height and earliness was competitive with the local taller and later-maturing material, thus expanding the diversity and genetic base for breeding. Material that combined positive characters for Northern Kazakhstan included cultivars and breeding lines from Russia (Don and National Grain Centers, Siberian breeding programs) and the USA (Kansas, Montana and Nebraska). Germplasm from Türkiye and Bulgaria also possessed valuable traits. The clear advantage of locally developed Kazakh material under severe winter kill suggests that crossing programs need to include one local parent to maintain high adaptation to ensure crop survival.
3. The superior genetic resources identified in the study combined high winter survival with productivity at Northern and Southern sites and resistance to stripe and leaf rust, WSMV. They have diverse geographical origins and pedigrees. Subject to germplasm restrictions, some may become variety candidates and be potentially released and cultivated. The study also built a valuable parental pool to be used in the improvement of this crop. Utilization of modern phenomics and genomic tools, along with speed breeding, would be essential for a winter wheat enhancement strategy for the region. The study also emphasizes the value of germplasm exchange and sharing as an important factor in reaching genetic gains.

## Figures and Tables

**Table 1:** Average values of agronomic traits in winter wheat trials across all sites, 2024–26.

Institution	Season	Average Values for the Traits:						
		WS, %	DH * from 1 May	Plant Height, cm	Stripe Rust, %	Leaf Rust, %	TKW, g	Grain Yield, g/m^2^
Kaz. RPC Grain	2024	72.9 ± 1.5	42.4 ± 0.3	80.9 ± 1.2	0	0	42.4 ± 0.4	558 ± 13
	2025	65.6 ± 1.7	-	-	0	0	-	-
	2026	64.1 ± 1.0	33.0 ± 0.2	80.6 ± 1.1	0	0	38.7 ± 0.4	476 ± 11
Kaz. RIAPG	2024	100	22.8 ± 0.3	93.6 ± 1.1	47.3 ± 0.4	9.8 ± 0.4	-	-
	2025	84.6 ± 1.3	14.7 ± 0.2	88.7 ± 1.4	12.9 ± 0.3	0	42.3 ± 0.4	632 ± 10
	2026	100	15.5 ± 0.4	92.2 ± 1.2	11.4 ± 0.4	6.0 ± 0.2	34.6 ± 0.4	418 ± 7
Karabalyk AES	2024	93.6 ± 0.9	34.9 ± 0.1	76.9 ± 0.9	0	51.9 ± 0.4	32.8 ± 0.4	-
	2025	47.1 ± 1.5	-	-	0	40–50 **	-	-
	2026	81.9 ± 1.6	35.0 ± 0.1	73.1 ± 1.2	0	40–50 **	37.6 ± 0.3	451 ± 12
North Kaz. AES	2024	72.8 ± 1.2	43.7 ± 0.2	-	0	9.4 ± 0.3	-	-
	2026	16.9 ± 2.0	38.9 ± 0.2	42.3 ± 0.8	0	0	31.4 ± 0.3	11.2 ± 2.0

*—DH–days to heading, **—leaf rust infection at the station’s winter wheat commercial fields; nursery evaluation was not conducted.

**Table 2:** Pearson correlation coefficients between grain yield and adaptation traits at individual trials, 2024–26.

Institution	Year	Coefficients of Correlation Between Grain Yield and:		
		Winter Survival	Days to Heading	Plant Height
Kaz. RPC Grain	2024	0.37 ***	−0.36 ***	0.31 ***
	2026	0.45 ***	0.07	0.17 *
Kaz. RIAPG	2025	0.23 **	−0.21 **	−0.03
	2026	-	−0.36 ***	−0.15
Karabalyk AES	2026	0.72 ***	−0.17 *	0.32 ***
North Kaz. AES	2026	0.61 ***	−0.13	0.45 ***

*, **, and ***—significant at probability levels 0.05, 0.01 and 0.001.

**Table 3:** Winter survival (%) of germplasm with different vernalization requirements across three Northern sites, 2024–26.

Vernalization Group	Number of Entries	Winter Survival (%) in Trials with Average Overwintering *:				Distribution (%) of the Germplasm from Different Breeding Programs ** in Vrn Groups:			
		>80%	60–80%	<50%	All	N. Kaz + Siberia	Russia—South	Türkiye	USA—Mid-West
Vrn(0)-35–70	7	79.2	65.8	21.5 ^b^	61.2	0	3.5	10.4	2.2
Vrn(20)-30–50	23	85.2	71.4	29.7 ^b^	65.6	25.0	0	17.3	17.8
Vrn(20)-51–70	83	87.0	70.3	29.3 ^b^	66.1	32.1	55.2	58.7	57.8
Vrn(20)-71–100	26	86.8	73.2	39.8 ^a^	69.3	32.1	24.1	6.8	15.5
Vrn(40)-35+	14	90.3	65.3	42.4 ^a^	68.7	10.8	17.2	6.8	6.7
Significance ***	-	n.s.	n.s	0.04	n.s.	-	-	-	-

*—values designated with different letters are significantly different at *p* < 0.05. **—N. Kaz + Siberia—Karabalyk AES, Omsk SAU and all Siberian programs; Russia—South—Don Center and National Grain Center; Türkiye—all programs; USA—Mid-West—Kansas, Oklahoma, Colorado and Nebraska, *** n.s. – not significant.

**Table 4:** Adaptation, disease resistance and productivity traits of winter wheat germplasm from different geographical regions, 2024–26.

**Trait**	Environments	Average Values of Agronomic Traits per Germplasm Origin Groups:					
		North Kaz. + Siberia	South Russia	Bulgaria	Türkiye	USA Mid-West	USA North-West
Number of entries		27	29	8	28	44	12
WS, %	All * (8 trials)	70.4 ± 1.1 ^a^ **	69.1 ± 1.0 ^ab^	65.0 ± 2.0 ^b^	60.2 ± 1.1 ^c^	67.3 ± 0.8 ^b^	67.3 ± 1.6 ^ab^
	N-Kaz. AES-26	46.5 ± 3.7 ^a^	18.8 ± 3.6 ^b^	1.2 ± 6.9 ^cd^	1.5 ± 3.7 ^d^	9.6 ± 2.9 ^bcd^	18.4 ± 5.6 ^bc^
DH, May 1	All * (8 trials)	32.1 ± 0.3 ^a^	30.3 ± 0.3 ^b^	29.7 ± 0.6 ^bc^	29.9 ± 0.3 ^b^	29.0 ± 0.3 ^c^	32.7 ± 0.5 ^a^
Plant height, cm	All * (7 trials)	98.2 ± 1.6 ^a^	76.5 ± 1.6 ^c^	78.4 ± 3.0 ^bc^	83.7 ± 1.6 ^b^	80.4 ± 1.3 ^bc^	83.1 ± 2.4 ^b^
Stripe rust, %	Kaz. RIAPG-24	65.5 ± 6.4 ^a^	35.2 ± 5.2 ^cd^	55.7 ± 10.5 ^abc^	46.2 ± 5.2 ^bc^	52.6 ± 4.2 ^ab^	20.4 ± 8.0 ^d^
Leaf rust, %	Karab. AES-24	64.8 ± 4.2 ^a^	45.0 ± 4.1 ^b^	26.9 ± 7.8 ^c^	63.4 ± 4.2 ^a^	43.9 ± 3.3 ^b^	45.4 ± 6.4 ^bc^
WSMV, %	Kaz. RPC Grain—26	23.3 ± 2.7 ^bc^	24.5 ± 2.6 ^bc^	30.0 ± 4.9 ^abc^	35.4 ± 2.6 ^a^	29.7 ± 2.1 ^ab^	20.3 ± 4.0 ^c^
Spike length, cm	All * (6 trials)	9.7 ± 0.2 ^a^	8.8 ± 0.2 ^b^	8.8 ± 0.3 ^b^	8.8 ± 0.2 ^b^	8.0 ± 0.1 ^d^	8.4 ± 0.2 ^c^
Spikelets/spike	All * (6 trials)	19.3 ± 0.2 ^a^	18.0 ± 0.2 ^b^	17.9 ± 0.4 ^b^	17.8 ± 0.3 ^b^	15.9 ± 0.1 ^c^	17.7 ± 0.2 ^b^
Grains/spike	All * (6 trials)	46.1 ± 0.7 ^a^	43.1 ± 0.8 ^b^	45.1 ± 1.9 ^ab^	43.4 ± 1.1 ^b^	39.7 ± 0.6 ^c^	44.3 ± 0.7 ^b^
Grain weight/spike, g	All * (6 trials)	1.89 ± 0.04 ^a^	1.77 ± 0.04 ^b^	1.91 ± 0.09 ^a^	1.87 ± 0.05 ^a^	1.45 ± 0.02 ^c^	1.49 ± 0.04 ^c^
TKW, g	All * (6 trials)	38.1 ± 0.5 ^c^	39.4 ± 0.4 ^b^	41.2 ± 1.2 ^a^	40.7 ± 0.4 ^a^	35.8 ± 0.4 ^d^	33.0 ± 0.7 ^e^

*—data from North-Kazakhstan AES in 2026 with high winter kill is not included in the mean calculation. **—means followed by the same letter within a row are not significantly different (Fisher’s protected LSD, *p* = 0.05) based on a one-way ANOVA of individual genotype values by germplasm group of origin (unequal-n LSD; error d.f. and MSE computed per trait/environment from all genotypes).

**Table 5:** Winter survival and grain yield stability parameters for the 10 top performers for each trait across all sites, 2024-26.

Entry #	Line/Variety	Origin	Mean	CV %	Pi *	Bi *	R^2^ *
Winter Survival, %							
4	Gerda	Local check-4	75.1	31.0	22.2	0.32	0.10
54	BC2F2 U346(113/96)/D//D/3/D	Omsk SAU	89.5	11.4	8.8	0.13	0.09
58	KP-134-18	Siberia	81.5	21.6	16.1	0.11	0.02
26	Stanichnaya	Nat. Grain Center	81.2	19.5	14.3	0.66	0.91
46	L.6545 k 3	Nat. Grain Center	78.7	27.3	18.2	0.76	0.65
23	Rostovchanka 7	Don Center	78.6	38.1	22.4	1.17	0.80
22	Konkurent	Nat. Grain Center	77.0	24.2	18.4	0.62	0.57
147	Panhandle	USA-UNL	76.9	39.5	22.9	1.22	0.84
104	KS13DH0039-99	USA-KSU	76.8	36.7	21.7	1.15	0.87
24	Donskaya yubileynaya	Don Center	76.5	34.3	20.9	1.10	0.91
116	KS13DH0021-136	USA-KSU	76.0	37.5	22.7	1.09	0.76
	LSD 0.05		16.4	-	-	-	-
Grain Yield, g/m^2^							
4	Gerda	Local check-4	450	43.5	213	0.77	0.73
108	KS14DH0024-28	USA-KSU	560	56.1	111	1.43	0.97
50	Zhiva	Nat. Grain Center	547	61.4	135	1.38	0.79
13	Lutescens 748-95	Karabalyk AES	535	47.9	151	1.13	0.92
121	Snowmass 2.0	USA-CSU	529	52.5	132	1.25	0.96
150	NE17544-2	USA-UNL	513	55.9	155	1.28	0.94
117	KS13DH0030-29	USA-KSU	512	59.0	212	1.08	0.60
40	L.7071 k 24	Nat. Grain Center	511	53.6	142	1.21	0.92
106	KS14DH0012-12	USA-KSU	509	64.8	176	1.35	0.79
107	KS14DH0017-1	USA-KSU	506	56.2	155	1.29	0.96
34	Svarog	Nat. Grain Center	505	62.4	180	1.14	0.61
	LSD 0.05		136	-	-	-	-

*—Pi—Lin and Binns [19] superiority measure scores each line by its average squared deviation from the best-performing entry; lower values mean a line stayed consistently close to the top performer; Bi—Eberhart and Russell [20] regression slope describes how a line’s performance responds to overall environmental quality; R^2^, the coefficient of determination of the regression line showing how much of the entry’s environment-to-environment variation is explained by that straight-line trend.

**Table 6:** Location of experimental sites and long-term weather parameters.

Institution	Location Name	Coordinates		Long-Term Air Temperature, °C		Long-Term Precipitation, mm	
		North	East	January	May–June	December–February	Year
Kaz. RPC Grain	Shortandy	51.631622	71.031765	−16.9	16.2	60.2	315.3
Kaz. RIAPG	Almalybak	43.232808	76.703217	−6.8	19.6	150.2	663.1
Karabalyk AES	Karabalyk	53.844492	62.130967	−15.9	18.3	64.2	380.1
North Kaz. AES	Shagalaly	54.165411	69.553617	−16.2	16.1	66.3	424.2

**Table 7:** Plot size, replication and summary of the traits recorded in the study, 2024–26.

Institution	Season	Plot Size, m^2^	Replication	Traits Recorded *:					
				Winter Survival	Days to Heading	Plant Height	Diseases	Spike Productivity	Grain Yield
Kaz. RPC Grain	2024	1.05	1	+	+	+	−	+	+
	2025	3.15	1	+	−	−	−	−	−
	2026	4.20	2	+	+	+	−	+	+
Kaz. RIAPG	2024	0.9	1	+	+	+	+	+	−
	2025	2.7	1	+	+	+	+	+	+
	2026	4.5	1	+	+	+	+	+	+
Karabalyk AES	2024	0.9	1	+	+	+	+	+	−
	2025	2.7	1	+	−	−	−	−	−
	2026	3.6	2	+	+	+	+	−	+
North Kaz. AES	2024	1.05	1	+	+	−	+	−	−
	2026	5.5	1	+	+	+	−	+	+

* “+” and “− “ refer to evaluation or non-evaluation of respective traits.

**Table 8:** Average monthly and long-term air temperature and precipitation for winter wheat seasons in 2023–2026.

Month	Kaz. RPC Grain		Kaz. RIAPG		Karabalyk AES		North-Kaz. AES	
	2023–26	Long-Term	2023–26	Long-Term	2023–26	Long-Term	2023–26	Long-Term
Air temperature, °C								
December	−9.6	−13.8	−1.2	−3.1	−10.3	−12.0	−10.5	−13.3
January	−13.6	−16.9	−2.4	−6.8	−14.0	−15.9	−15.1	−16.2
February	−14.1	−14.5	−1.1	−4.6	−13.1	−13.8	−13.8	−15.6
March	−5.4	−6.2	5.5	4.6	−3.4	−5.1	−4.7	−6.4
April	9.0	5.5	14.6	12.5	10.3	7.1	9.1	6.0
May	15.1	13.6	18.8	17.2	14.7	15.4	14.1	13.6
June	22.0	18.8	25.1	22.1	21.8	21.0	21.1	18.7
July	20.8	20.0	26.3	24.9	23.0	22.3	20.9	20.1
Season	3.0	0.8	10.7	8.4	3.5	2.4	2.5	0.9
Precipitation, mm								
December	22.3	30.0	54.0	41.0	39.7	23.0	27.0	26.0
January	29.0	20.0	37.0	36.0	29.0	21.0	21.0	21.0
February	23.0	18.0	44.7	44.0	23.0	21.0	19.9	19.0
March	19.0	22.0	88.0	81.0	7.7	22.0	16.3	22.0
April	9.7	21.0	91.7	107.0	31.3	25.0	12.6	30.0
May	33.7	26.0	118.0	95.0	82.0	36.0	39.3	32.0
June	37.0	36.0	23.3	53.0	73.0	42.0	72.7	51.0
July	43.3	55.0	46.1	40.0	61.8	64.0	81.7	76.0
Season	217.0	228.0	504.2	497.0	356.7	254.0	296.4	277.0

## Data Availability

The data is available from the authors upon request, subject to restrictions by the germplasm originating institutions. Most of the germplasm shared is subject to Material Transfer Agreements, which commonly follow the SMTA (https://www.fao.org/plant-treaty/areas-of-work/the-multilateral-system/smta/en/ accessed 7 September, 2026), with some specifics depending on institutional rules and regulations.

## Supplementary Materials

The following supporting information can be downloaded at https://www.mdpi.com/article/10.3390/plants15213258/s1, Figure S1: Average winter survival of the germplasm across four sites in 2024–26; Figure S2: Biplot of winter survival, number of days to heading and plant height at eight trials across three Northern sites in 2024–2026; Figure S3: Relationship between WSMV severity and grain yield at Kaz. RPC Grain, 2026; Table S1: ANOVA significance of the effects of genotypes, locations and their interaction in combined analysis of 2026 trials at Kaz. RPC Grain and Karabalyk AES; Table S2: Pearson coefficients of correlation between winter survival and days to heading and plant height on 149 genotypes across Northern study sites in 2024–2026; Table S3: Productivity of winter wheat germplasm with different origins, 2024–26; Table S4: Winter survival and grain productivity stability parameters for 30 top performers for each trait, 2024–26; Table S5: Winter survival for top 20 best genotypes and grain yield at North Kazakhstan AES, 2026; Table S6: Winter survival and grain productivity of 20 highest yielding genotypes at Kaz. RPC Grain and Karabalyk AES in 2026; Table S7: Stripe and leaf rust severity of resistant genotypes, 2024–26; Table S8: WSMV severity of resistant genotypes, Kaz. RPC Grain, 2026; Table S9: Winter wheat material used in the study; Table S10: Experimental design description used in the study, 2024–26; Table S11: Average monthly and seasonal temperature and precipitation at the experimental sites in 2023–26; Table S12: Trait adjustment in unreplicated trials using check-plot correction.

## Abbreviations

The following abbreviations are used in this manuscript:

TKW	1000-kernel weight
AES	Agricultural Experimental Station
Kaz. RPC Grain	Kazakh Research–Production Center of Grain
Kaz. RIAPG	Kazakh Research Institute of Agriculture and Plant Growing
RGBD	Randomized Complete Block Design
SAU	State Agrarian University
WS	Winter survival
