# Supplementary material for: Genetic Resources for Winter Wheat Cultivation Under Severe Cold in Northern Kazakhstan
Source: Plants (Basel). 2026 Oct 22;15(21):3258. doi: 10.3390/plants15213258 (PMC13765624; doi:10.3390/plants15213258)
Supplement: Supplementary file 1 [file plants-15-03258-s001.zip › plants-4633572-supplementary.pdf]

Table S1. ANOVA significance of the effects of genotypes, locations and their interaction in combined analysis of 2026 trials at Kaz. RPC Grain and Karabalyk AES.

**Trait**	**P values of the effects of:**		
	**Locations**	**Genotypes**	**Location x genotypes**
Days to heading	0.0333*	<0.001***	<0.001***
Plant height	<0.001***	<0.001***	<0.001***
Grain yield	n.s.	<0.001***	<0.001***

Table S2. Pearson coefficients of correlation of winter survival with days to heading and plant height on 149 genotypes across Northern study sites in 2024-2026.

Institution	Year	Coefficients of correlation between winter survival and:	
		Days to heading [a]	Plant height [a]
Kaz. RPC Grain	2024	-0.12	0.05
	2025	-0.06	-0.10
	2026	-0.14	0.03
Karabalyk AES	2024	-0.27**	0.18*
	2025	0.09	0.06
	2026	-0.04	0.26**
North Kaz. AES	2024	-0.56***	0.03
	2026	0.36***	0.43***

[a] – in the absence of data for heading and plant height for specific season – average values over three years were taken for calculations.

*, **, *** - significant at probability level 0.05; 0.01 and 0.001, respectively.

Table S3. Productivity of winter wheat germplasm with different origin, 2024-26.

Trait	Environments	Average values of grain yield (g/m²) per germplasm origin groups:					
		**North Kaz. + Siberia**	**South Russia**	**Bulgaria**	**Türkiye**	**USA mid-West**	**USA North-West**
Number of entries		27	29	8	28	44	12
Yield, g/m²	All* (5 trials)	521±11ab**	536±11a	482±21bc	450±11c	516±9ab	524±17ab
	Kaz. RIAPG-25-26	507±15b	515±14b	474±27b	522±15ab	558±11.6a	508±22b
	2 northern sites*, 24-26	530±17a	551±16a	488±31ab	398±16c	486±13b	536±25ab

* - data from North-Kazakhstan AES in 2026 with high winter kill is not included into mean calculation.

** - means followed by the same letter within a row are not significantly different (Fisher's protected LSD, P = 0.05), based on a one-way ANOVA of individual genotype values by germplasm group of origin (unequal-n LSD; error d.f. and MSE computed per trait/environment from all genotypes).

Table S4. Winter survival and grain productivity stability parameters for 30-top performers for each trait, 2024-26.

Entry #	Line / variety	Origin group	Mean	CV %	Pi	bi	R^2
**Winter survival, %**							
1	Steklovidnaya-24	Local check-1	51.4	53.0	36.6	0.82	0.47
2	Vavilov	Local check-2	52.5	65.4	38.2	0.94	0.39
3	Skipetr	Local check-3	72.5	19.0	19.6	0.30	0.25
4	Gerda	Local check-4	75.1	31.0	22.2	0.32	0.10
54	BC2F2 U346(113x96)xDxDxD	North Kaz. + Siberia	89.5	11.4	8.8	0.13	0.09
58	KP-134-18	North Kaz. + Siberia	81.5	21.6	16.1	0.11	0.02
26	STANICHNAYA	Russia-South	81.2	19.5	14.3	0.66	0.91
46	L.6545 к 3	Russia-South	78.7	27.3	18.2	0.76	0.65
23	ROSTOVCHANKA 7	Russia-South	78.6	38.1	22.4	1.17	0.80
22	KONKURENT	Russia-South	77.0	24.2	18.4	0.62	0.57
147	Panhandle	USA-Mid-West	76.9	39.5	22.9	1.22	0.84
104	KS13DH0039-99	USA-Mid-West	76.8	36.7	21.7	1.15	0.87
24	DONSKAYA YUBILEYNAYA	Russia-South	76.5	34.3	20.9	1.10	0.91
116	KS13DH0021-136	USA-Mid-West	76.0	37.5	22.7	1.09	0.76
126	MT WarCat	USA-North-West	75.8	36.9	22.2	0.87	0.50
40	L.7071 к 24	Russia-South	74.1	40.8	24.8	0.97	0.54
123	Guardian (Wcm + Wsm2)	USA-Mid-West	73.8	29.6	21.1	0.61	0.41
59	Izaura	North Kaz. + Siberia	73.8	30.3	21.4	0.42	0.19
55	BC1F2 U361(113x96)xDxD	North Kaz. + Siberia	73.7	27.3	19.9	0.82	0.86
127	MT1745	USA-North-West	73.5	31.0	21.3	0.60	0.36
67	Omskaya 4	North Kaz. + Siberia	73.3	26.5	20.4	0.59	0.48
110	KS100028K-11	USA-Mid-West	73.2	43.0	25.0	1.11	0.65
66	Omskaya ozimaya	North Kaz. + Siberia	72.8	32.0	21.9	0.95	0.87
148	Freeman	USA-Mid-West	72.3	32.6	22.7	0.61	0.35
62	Erithrospermum 956	North Kaz. + Siberia	72.1	42.8	25.5	1.22	0.81
25	ZERNOGRADKA11	Russia-South	72.1	31.1	22.0	0.65	0.44
131	Warhorse	USA-North-West	72.0	37.6	24.2	1.01	0.73

64	KSI 35/20	North Kaz. + Siberia	72.0	32.9	23.1	0.77	0.55
114	KS13DH0013-112	USA-Mid-West	71.8	49.9	27.8	1.44	0.84
13	Lutescens 748-95	North Kaz. + Siberia	71.8	32.2	23.3	0.41	0.16
5	Zere	North Kaz. + Siberia	71.8	34.9	24.8	0.35	0.10
35	ETNOS	Russia-South	71.8	38.2	24.4	1.11	0.85
134	OK20D78S014	USA-Mid-West	71.8	39.6	24.5	1.23	0.97
112	KS13DH0007-20	USA-Mid-West	71.7	29.6	21.5	0.87	0.87
**Grain yield, g/m2**							
1	Steklovidnaya-24	Local check-1	282	93.5	352	0.74	0.37
2	Vavilov	Local check-2	283	104.1	355	0.85	0.39
3	Skipetr	Local check-3	435	63.0	221	0.96	0.58
4	Gerda	Local check-4	450	43.5	213	0.77	0.73
108	KS14DH0024-28	USA-Mid-West	560	56.1	111	1.43	0.97
50	Zhiva	Russia-South	547	61.4	135	1.38	0.79
13	Lutescens 748-95	North Kaz. + Siberia	535	47.9	151	1.13	0.92
121	Snowmass 2.0 (Wsm2)	USA-Mid-West	529	52.5	132	1.25	0.96
150	NE17544-2	USA-Mid-West	513	55.9	155	1.28	0.94
117	KS13DH0030-29	USA-Mid-West	512	59.0	212	1.08	0.60
40	L.7071 к 24	Russia-South	511	53.6	142	1.21	0.92
106	KS14DH0012-12	USA-Mid-West	509	64.8	176	1.35	0.79
107	KS14DH0017-1	USA-Mid-West	506	56.2	155	1.29	0.96
34	SVAROG	Russia-South	505	62.4	180	1.14	0.61
129	MTFH20170	USA-North-West	503	53.1	153	1.20	0.94
56	OmGAU №5	North Kaz. + Siberia	502	59.0	171	1.30	0.91
7	Lutescens 436H60-1	North Kaz. + Siberia	500	48.0	186	1.01	0.84
12	Lutescens -SP-2-220	North Kaz. + Siberia	498	53.9	179	1.16	0.88
41	L.7069 к 1	Russia-South	496	52.4	159	1.12	0.87
45	L.6471 к 13	Russia-South	493	52.1	171	1.11	0.89
122	Snowmass (Wsm2)	USA-Mid-West	492	52.3	172	1.16	0.96
87	SVILENA/4/PEHL//RPB 8-68/CHRC/3/506/88-113	Türkiye	491	53.1	175	1.01	0.70

127	MT1745	USA-North-West	489	57.0	170	1.17	0.83
9	Lutescens SP-1-3269	North Kaz. + Siberia	487	49.1	196	0.99	0.80
47	L.6509 к 6	Russia-South	485	59.1	168	1.24	0.88
151	NE18445	USA-Mid-West	485	48.6	180	1.06	0.95
37	L.6371 к 4	Russia-South	484	57.5	181	1.03	0.65
118	KS13DH002722	USA-Mid-West	483	51.1	173	1.11	0.95
124	JUDEE	USA-North-West	483	49.5	190	1.02	0.85
110	KS100028K-11	USA-Mid-West	483	58.3	175	1.25	0.93
132	Breakthrough	USA-Mid-West	480	61.0	199	1.21	0.81
119	KS13DH0030-32	USA-Mid-West	478	58.6	181	1.25	0.94
26	STANICHNAYA	Russia-South	477	50.8	171	1.11	0.99
22	KONKURENT	Russia-South	475	59.9	132	1.19	0.93

Table S5. Winter survival for top 20 best genotypes and grain yield at North Kazakhstan AES, 2026.

Entry #	Cultivar/line	Origin	Winter survival, %	Grain yield, g/m²
4	Gerda	Local check-4	80	124
3	Skipetr	Local check-3	60	83
58	KP-134-18	Siberia	90	0
8	Albidum KP-59-60	Karabalyk AES	90	64
54	BC2F2 U346(113x96)xDxDxD	Omsk SAU	90	18
9	Lutescens SP-1-3269	Karabalyk AES	85	71
11	Lutescens SP-1-6217	Karabalyk AES	80	12
59	Izaura	Siberia	80	55
13	Lutescens 748-95	Karabalyk AES	70	110
5	Zere	Karabalyk AES	70	75
10	Lutescens KP-11-93	Karabalyk AES	70	73
127	MT1745	USA-MSU	65	6
36	L.6170 к 22	Nat. Grain Center	65	8
123	Guardian	USA-CSU	60	0
12	Lutescens -SP-2-220	Karabalyk AES	60	47
25	Zernogradka 11	Don Center	60	47
148	Freeman	USA-UNL	60	38
67	Omskaya 4	Siberia	60	25
27	Asket	Don Center	55	11
45	L.6471 к 13	Nat. Grain Center	50	7
7	Lutescens 436H60-1	Karabalyk AES	50	79
37	L.6371 к 4	Nat. Grain Center	50	16

Table S6. Winter survival and grain productivity of 20 highest yielding genotypes at Kaz. RPC Grain and Karabalyk AES in 2026.

Entry #	Cultivar, line	Origin	Winter survival, %			Grain yield, g/m2		
			Kaz. RPC Grain	Karabalyk AES	Average	Kaz. RPC Grain	Karabalyk AES	Average
1	Steklovidnaya-24	Check-1	65	78	72	385	290	337
2	Vavilov	Check-2	40	10	25	500	0	250
3	Skipetr	Check-3	85	80	83	340	478	409
4	Gerda	Check-4	88	100	94	555	588	571
117	KS13DH0030-29	US-KSU	85	75	80	781	622	702
146	Robidoux	USA-UNL	95	88	91	725	609	667
34	Svarog	RUS-NGC	83	98	90	608	702	655
45	L.6471 к 13	RUS-NGC	90	95	93	711	548	629
9	Lutescens SP-1-3269	Karab. AES	93	88	90	603	650	626
22	Konkurent	RUS-Don	88	90	89	669	577	623
123	Guardian	USA-CSU	85	95	90	593	642	618
28	Donna	RUS-Don	83	80	81	609	618	614
124	Judee	USA-MSU	98	73	85	669	546	608
129	MTFH20170	USA-MSU	93	93	93	671	527	599
64	KSI 35-20	RUS-Siberia	90	100	95	511	676	594
131	Warhorse	USA-MSU	93	93	93	651	527	589
147	Panhandle	USA-UNL	90	95	93	567	608	587
127	MT1745	USA-MSU	93	98	95	536	636	586
65	KSI 34-21	RUS-Siberia	95	98	96	545	626	586
118	KS13DH002722	US-KSU	88	93	90	637	523	580
121	Snowmass 2.0	USA-CSU	80	85	83	545	613	579
7	Lutescens 436H60-1	Karab. AES	83	95	89	519	638	578
62	Erithrospermum 956	Siberia	90	93	91	520	634	577
87	Svilena/4/…113	TUR-Konya	88	83	85	624	522	573
	LSD 0.05		31	18	22	196	185	130

Table S7. Stripe and leaf rust severity of resistant genotypes, 2024-26.

Entry #	Line / variety	Origin group	Highest severity %	Mean severity %
**Stripe rust, % (Kaz. RIAPG, 2024-26)**				
1	Steklovidnaya-24 (LC-1)		60	33.3
44	L.7012 к 7	Russia-South	0	0.0
98	LCS JET	USA-North-West	0	0.0
109	KS14DH0024-61	USA-Mid-West	0	0.0
140	OK21P724	USA-Mid-West	0	0.0
153	NE14434	USA-Mid-West	0	0.0
101	OR2050331H/2*CO01W171-t	USA-North-West	5	1.7
69	ATTILA*2/PBW65/YAKAR	Türkiye	5	1.7
41	L.7069 к 1	Russia-South	5	3.3
114	KS13DH0013-112	USA-Mid-West	5	1.7
141	OK21910	USA-Mid-West	5	1.7
57	OmGAU №10	North Kaz. + Siberia	5	1.7
115	KS13DH0020-59	USA-Mid-West	5	1.7
40	L.7071 к 24	Russia-South	10	6.7
77	GP 2558-128/2*Pliska//Ekiz	Türkiye	10	3.3
68	NACIBEY	Türkiye	10	3.3
43	L.7013 к 18	Russia-South	10	3.3
90	CANADIAN/CUNNINGHAM//…/PASTOR	Türkiye	10	6.7
129	MTFH20170	USA-North-West	10	5.0
118	KS13DH002722	USA-Mid-West	10	3.3
48	L.5730 к 4-3	Russia-South	10	3.3
142	(ATTILA*2/ESDA//…KS91W047	USA-Mid-West	10	5.0
42	L.7059 к 6	Russia-South	10	3.3
50	Zhiva	Russia-South	10	3.3
7	Lutescens 436H60-1	North Kaz. + Siberia	15	12.5
131	Warhorse	USA-North-West	15	5.0
95	MOMTCHILL/2*GUN91//NACIBEY	Türkiye	15	8.3
120	Langin (Wcm)	USA-Mid-West	15	6.7
10	Lutescens KP-11-93	North Kaz. + Siberia	20	20.0
38	L.4620 к 2-1-11	Russia-South	20	10.0
6	Lutescens KP-320-93	North Kaz. + Siberia	20	10.0
11	Lutescens SP-1-6217	North Kaz. + Siberia	20	15.0
85	DAMLA	Türkiye	20	6.7
82	SG-U 7067/5/TEMU39.76/..../4/KK8514.1	Türkiye	20	6.7
125	Northern	USA-North-West	20	13.3
128	MTS2068	USA-North-West	20	6.7
126	MT WarCat	USA-North-West	20	6.7
110	KS100028K-11	USA-Mid-West	20	6.7
99	WHETSTONE	USA-North-West	20	6.7
100	ORN00B553/SD01W064//… PALOMINO	USA-North-West	20	10.0
108	KS14DH0024-28	USA-Mid-West	20	6.7

130	MT2225	USA-North-West	20	6.7
37	L.6371 к 4	Russia-South	20	10.0
46	L.6545 к 3	Russia-South	20	11.7
47	L.6509 к 6	Russia-South	20	6.7
22	KONKURENT	Russia-South	20	10.0
**Leaf rust, % (Kaz. RIAPG, 2024, 2026; Karabalyk AES, 2024; North-Kazakhstan AES, 2024)**				
1	Steklovidnaya-24 (LC-1)		100	53.8
14	MERILIN	Bulgaria	5	1.5
115	KS13DH0020-59	USA-Mid-West	5	1.3
108	KS14DH0024-28	USA-Mid-West	5	1.3
15	LAZALKA	Bulgaria	10	2.8
42	L.7059 к 6	Russia-South	10	2.5
107	KS14DH0017-1	USA-Mid-West	10	2.5
34	SVAROG	Russia-South	10	2.5
37	L.6371 к 4	Russia-South	10	2.5
111	KS100298K-9	USA-Mid-West	10	2.5
40	L.7071 к 24	Russia-South	25	6.5
45	L.6471 к 13	Russia-South	25	12.5
50	Zhiva	Russia-South	25	6.3
39	m. K5	Russia-South	25	6.3
26	STANICHNAYA	Russia-South	25	6.5
27	ASKET	Russia-South	25	6.5
16	PCELINA	Bulgaria	25	6.5
113	KS13DH0012-4	USA-Mid-West	25	6.3
114	KS13DH0013-112	USA-Mid-West	25	6.5
112	KS13DH0007-20	USA-Mid-West	25	6.3
110	KS100028K-11	USA-Mid-West	25	6.3
106	KS14DH0012-12	USA-Mid-West	25	6.3
109	KS14DH0024-61	USA-Mid-West	25	6.5
105	KS13DH0053-32	USA-Mid-West	25	6.3
104	KS13DH0039-99	USA-Mid-West	25	6.5
101	OR2050331H/2*CO01W171-t	USA-North-West	25	8.8
79	YUKSEL	Türkiye	25	6.3
72	WBLL1*2/TUKURU//BILLINGS	Türkiye	25	6.5
73	MV233-05//.../3/GONDVANA	Türkiye	25	11.5
18	GTW KORONA	Bulgaria	25	6.5
25	ZERNOGRADKA11	Russia-South	25	6.3
119	KS13DH0030-32	USA-Mid-West	25	6.3
118	KS13DH002722	USA-Mid-West	25	7.8
116	KS13DH0021-136	USA-Mid-West	25	6.3
132	Breakthrough	USA-Mid-West	25	8.8
124	JUDEE	USA-North-West	25	10.0
123	Guardian (Wcm + Wsm2)	USA-Mid-West	25	13.8
117	KS13DH0030-29	USA-Mid-West	25	7.8
153	NE14434	USA-Mid-West	25	6.3
151	NE18445	USA-Mid-West	25	12.8
143	OK01307/DUSTER//OK06822W	USA-Mid-West	25	6.3

Table S8. WSMV severity of resistant genotypes, Kaz. RPC Grain, 2026.

Entry #	Line / variety	Origin group	WSMV, %
2	Vavilov	Local check	
129	MTFH20170	USA-MSU	1
117	KS13DH0030-29	US-KSU	3
146	Robidoux	USA-UNL	5
131	Warhorse	USA-MSU	8
109	KS14DH0024-61	USA-KSU	8
144	OCW03S667T-2/KS020986~1	USA-OkSU	8
130	MT2225	USA-MSU	10
28	Donna	Don Center	10
58	KP-134-18	Siberia	10
8	Albidum KP-59-60	Karabalyk AES	10
45	L.6471 к 13	National Grain Center	10
7	Lutescens 436H60-1	Karabalyk AES	10
104	KS13DH0039-99	US-KSU	10
107	KS14DH0017-1	US-KSU	10
96	SY Wolf	USA	10

Table S9. Winter wheat material used in the study.

Entry #	Cultivar/line	Country origin	Breeding program origin*
1	Steklovidnaya-24 (LC-1)	KAZ	Kaz RIAPG
2	Vavilov (LC--2)	KAZ	Kaz RIAPG
3	Skipetr (LC-3)	RUS	RUS
4	Gerda (LC-4)	KAZ	Karabalyk AES
14	MERILIN	BUL	Bulgaria programs
15	LAZALKA	BUL	Bulgaria programs
16	PCELINA	BUL	Bulgaria programs
17	BOJANA	BUL	Bulgaria programs
18	GTW KORONA	BUL	Bulgaria programs
19	GTW DRAGANA	BUL	Bulgaria programs
20	GTP RADA	BUL	Bulgaria programs
21	GTP KALINA	BUL	Bulgaria programs
5	Zere	KAZ	Karabalyk AES
6	Lutescens KP-320-93	KAZ	Karabalyk AES
7	Lutescens 436H60-1	KAZ	Karabalyk AES
8	Albidum KP-59-60	KAZ	Karabalyk AES
9	Lutescens SP-1-3269	KAZ	Karabalyk AES
10	Lutescens KP-11-93	KAZ	Karabalyk AES
11	Lutescens SP-1-6217	KAZ	Karabalyk AES
12	Lutescens -SP-2-220	KAZ	Karabalyk AES
13	Lutescens 748-95	KAZ	Karabalyk AES
22	KONKURENT	RUS	Don Center
23	ROSTOVCHANKA7	RUS	Don Center
24	DONSKAYA YUBILEYNAYA	RUS	Don Center
25	ZERNOGRADKA11	RUS	Don Center
26	STANICHNAYA	RUS	Don Center
27	ASKET	RUS	Don Center

28	Donna	RUS	Don Center
29	DONERA	RUS	Don Center
30	DONSKOY MAYAK	RUS	Don Center
31	DON107	RUS	Don Center
32	DONSKOY SURPRIZ	RUS	Don Center
33	SAMBEK	RUS	Nat. Grain Center
34	SVAROG	RUS	Nat. Grain Center
35	ETNOS	RUS	Nat. Grain Center
36	L.6170 к 22	RUS	Nat. Grain Center
37	L.6371 к 4	RUS	Nat. Grain Center
38	L.4620 к 2-1-11	RUS	Nat. Grain Center
39	m. K5	RUS	Nat. Grain Center
40	L.7071 к 24	RUS	Nat. Grain Center
41	L.7069 к 1	RUS	Nat. Grain Center
42	L.7059 к 6	RUS	Nat. Grain Center
43	L.7013 к 18	RUS	Nat. Grain Center
44	L.7012 к 7	RUS	Nat. Grain Center
45	L.6471 к 13	RUS	Nat. Grain Center
46	L.6545 к 3	RUS	Nat. Grain Center
47	L.6509 к 6	RUS	Nat. Grain Center
48	L.5730 к 4-3	RUS	Nat. Grain Center
49	Vestnitsa	RUS	Nat. Grain Center
50	Zhiva	RUS	Nat. Grain Center
51	Donera/UKR-OD 1530.94/AE.SQUARROSA(392)	RUS	Omsk SAU
52	Donera/UKR-OD 1530.94/AE.SQUARROSA(392)	RUS	Omsk SAU
53	Donera/UKR-OD 1530.94/AE.SQUARROSA(392)	RUS	Omsk SAU
54	BC2F2 U346(113x96)xDxDxD	RUS	Omsk SAU
55	BC1F2 U361(113x96)xDxD	RUS	Omsk SAU
56	OmGAU №5	RUS	Omsk SAU
57	OmGAU №10	RUS	Omsk SAU

58	KP-134-18	RUS	Siberia programs
59	Izaura	RUS	Siberia programs
60	Erithrospermum 951	RUS	Siberia programs
61	Erithrospermum 955	RUS	Siberia programs
62	Erithrospermum 956	RUS	Siberia programs
63	Erithrospermum 977	RUS	Siberia programs
64	KSI 35/20	RUS	Siberia programs
65	KSI 34/21	RUS	Siberia programs
66	Omskaya ozimaya	RUS	Siberia programs
67	Omskaya 4	RUS	Siberia programs
69	ATTILA*2/PBW65/YAKAR	TUR	TCI
70	ATTILA/2*PASTOR//YUMAI 29	TUR	TCI
68	NACIBEY	TUR	TCI
71	Gondvana//HBK0935-29-15/KS90W077-2-2/VBF0589-1	TUR	TCI
72	WBLL1*2/TUKURU//BILLINGS	TUR	Turkey programs
73	MV233-05//PANTHEON/BLUEGIL-2/3/GONDVANA	TUR	Turkey programs
74	SABAN	TUR	Turkey programs
75	WBLL1*2/KURUKU/5/CHUEN-MAI-18/2137//2174/4/CTY*3/TA2460//2163/3/CRR (OK98610) (OCW00M797T-2)/6/TX91D6991/B1551	TUR	Turkey programs
76	KUV/LJILN//Oracle/Pehlivan	TUR	Turkey programs
77	GP 2558-128/2*Pliska//Ekiz	TUR	TCI
78	KOPRU	TUR	TUR-Edirne
79	YUKSEL	TUR	TUR-Edirne
80	3971-6 (BUL)/6/SIDERAL/3/BAU/LIRA//KAUZ/5/LOV26//LFN/SDY(ES84-24)/3/SERI/4/SERI	TUR	TUR-Edirne
81	3971-6 (BUL)/6/SIDERAL/3/BAU/LIRA//KAUZ/5/LOV26//LFN/SDY(ES84-24)/3/SERI/4/SERI	TUR	TUR-Edirne
82	SG-U 7067/5/TEMU39.76/CHAT//CUPE/3/M1223.3D.1D/ALD/4/KK8514.1	TUR	TUR-Edirne
83	PEHLIVAN	TUR	TUR-Edirne
84	GELIBOLU	TUR	TUR-Edirne
85	DAMLA	TUR	TUR-Edirne
86	HK206/5/OR F1.158/FDL//BLO/3/CA8055/4/KS82W409/SPN/6/KONYA 2002	TUR	TUR-Konya

87	SVILENA/4/PEHL//RPB 8-68/CHRC/3/506/88-113	TUR	TUR-Konya
88	KC 7-89-7 (BUL-KC)//854.87.1.13/SANA/4/MVM/SANA//PFAU/MILAN/3/BUL1518-4-38	TUR	TUR-Konya
89	MV17//KREMENA/LOV29/3/KATEA-1/4/SONMEZ01	TUR	TUR-Konya
90	CANADIAN/CUNNINGHAM//KENNEDY/3/AGRI/NAC//ATTILA/4/KS82142/PASTOR	TUR	TUR-Konya
91	ALTAY2000/4/VORONA//MILAN/SHA7/3/MV17/5/ALTAY2000	TUR	TUR-Konya
92	COOK/VEE//DOVE/SERI/3/GEN/GOLIA/4/ALTAY-2000	TUR	TUR-Konya
93	KATE-A-1/ONEARLY_S-115	TUR	TUR-Konya
94	SONMEZ01/CH75267//KATE-A1	TUR	TUR-Konya
95	MOMTCHILL/2*GUN91//NACIBEY	TUR	TUR-Konya
96	SY Wolf	USA	USA programs
97	L-386	USA	USA programs
98	LCS JET	USA	USA programs
99	WHETSTONE	USA	USA programs
120	Langin (Wcm)	USA	USA-CSU
121	Snowmass 2.0 (Wsm2)	USA	USA-CSU
122	Snowmass (Wsm2)	USA	USA-CSU
123	Guardian (Wcm + Wsm2)	USA	USA-CSU
102	KS13DH0030-29-1	USA	USA-KSU
103	KS13DH0030-33	USA	USA-KSU
104	KS13DH0039-99	USA	USA-KSU
105	KS13DH0053-32	USA	USA-KSU
106	KS14DH0012-12	USA	USA-KSU
107	KS14DH0017-1	USA	USA-KSU
108	KS14DH0024-28	USA	USA-KSU
109	KS14DH0024-61	USA	USA-KSU
110	KS100028K-11	USA	USA-KSU
111	KS100298K-9	USA	USA-KSU
112	KS13DH0007-20	USA	USA-KSU
113	KS13DH0012-4	USA	USA-KSU

114	KS13DH0013-112	USA	USA-KSU
115	KS13DH0020-59	USA	USA-KSU
116	KS13DH0021-136	USA	USA-KSU
117	KS13DH0030-29	USA	USA-KSU
118	KS13DH002722	USA	USA-KSU
119	KS13DH0030-32	USA	USA-KSU
100	ORN00B553/SD01W064//AGRIPRO PALOMINO	USA	USA-WSU
101	OR2050331H/2*CO01W171-t	USA	USA-WSU
124	JUDEE	USA	US-MSU
125	Northern	USA	US-MSU
126	MT WarCat	USA	US-MSU
127	MT1745	USA	US-MSU
128	MTS2068	USA	US-MSU
129	MTFH20170	USA	US-MSU
130	MT2225	USA	US-MSU
131	Warhorse	USA	US-MSU
132	Breakthrough	USA	US-OkSU
133	OK20D78S002	USA	US-OkSU
134	OK20D78S014	USA	US-OkSU
135	OK20D78S015	USA	US-OkSU
136	OK20D78S016	USA	US-OkSU
137	OK21913	USA	US-OkSU
138	OK21PS823	USA	US-OkSU
139	OK21917	USA	US-OkSU
140	OK21P724	USA	US-OkSU
141	OK21910	USA	US-OkSU
142	(ATTILA*2/ESDA//MASON)/(HBK0935-7-4/BETTY 'S')//KS91W047	USA	US-OkSU
143	OK01307/DUSTER//OK06822W	USA	US-OkSU
144	OCW03S667T-2/KS020986~1	USA	US-OkSU

145	Ruth	USA	US-UNL
146	Robidoux	USA	US-UNL
147	Panhandle	USA	US-UNL
148	Freeman	USA	US-UNL
149	NE17441	USA	US-UNL
150	NE17544-2	USA	US-UNL
151	NE18445	USA	US-UNL
152	NE18573	USA	US-UNL
153	NE14434	USA	US-UNL

* Kaz RIAPG – Kazakh Research Institute of Agriculture and Plant Growing, Almaty region, Kazakhstan; Karabalyk AES – Karabalyk Agricultural Experimental Station, Kostanay reg., Kazakhstan, Don Center – Don Breeding Program, Rostov region, Russia; Nat. Grain Center – National Grain Center, Krasnodar, Russia; Omsk SAU – Omsk State Agrarian University; TCI – Turkey-CIMMYT-ICARDA, TUR-Edirne – Thrace region Breeding program, Edirne, Turkey; TUR-Konya – Bahri Dagdas International Agric. Research Center, Konya, Turkey; USA-KSU – Kansas State University; US-MSU – Montana State University; US-OkSU – Oklahoma State University, US-UNL – University of Nebraska – Lincoln.

Table S10. Experimental design description used in the study, 2024-26.

Experimental design character	Kaz. RPC Grain			Kaz RIAPG			Karabalyk AES			North-Kazakhstan AES		
	2024	2025	2026	2024	2025	2026	2024	2025	2026	2024	2025	2026
Experimental unit	Plot	Plot	Plot	Plot	Plot	Plot	Plot	Plot	Plot	Plot	Plot	Plot
Planting date	09.09.23	19.09.24	5.09.25	05.10.23	03.10.24	09.10.25	05.09.23	19.09.24	18.09.25	12.09	15.09	27.08
Number of rows	7	7	7	6	6	10	6	6	6	7	Experiment failed	7
Plot dimension, m	1 x 1.05	3 x 1.05	4 x 1.05	1 x 0.9	3 x 0.9	3 x 1.5	1 x 0.9	3 x 0.9	4 x 0.9	1 x 1.05		5 x 1.05
Plot size, m2	1.05	3.15	4.20	0.9	2.7	4.5	0.9	2.7	3.6	1.05		5.5
Harvested area, m2	1.05	3.15	4.20	0.9	2.7	4.5	0.9	2.7	3.6	1.05		5.5
Randomization	None	None	RCBD	None	None	None	None	None	RCBD	None		None
Replication	1	1	2	1	1	1	1	1	2	1		1
Border dimensions, m	0.35 x 0.6	0.35 x 1.0	0.35 x 1.0	0.4 x 0.5	0.4 x 0.5	0.4 x 0.5	1 x 0.5	3 x 0.5	3 x 0.5	0.7 x 1.0		0.7 x 1.0
Frequency of Steklovidnaya-24 (Local check-1)	Every 30 entries	Every 50 entries	Every 25 entries	Every 30 entries	Every 50 entries	Every 25 entries	Every 30 entries	Every 50 entries	Every 25 entries	Every 30 entries	Every 50 entries	Every 25 entries

Table S11. Average monthly and seasonal temperature and precipitation at the experimental sites in 2023-26.

Month	Kaz. RPC Grain				Kaz RIAPG				Karabalyk AES				North-Kazakhstan AES			
	2023-24	2024-25	2025-26	L-t*	2023-24	2024-25	2025-26	L-t*	2023-24	2024-25	2025-26	L-t*	2023-24	2024-25	2025-26	L-t*
**Air temperature, °C**																
December	-9.9	-9.2	-9.8	-13.8	-1.0	-3.3	0.7	-3.1	-12.9	-7.6	-10.3	-12.0	-14.2	-6.8	-10.6	-13.3
January	-14.0	-11.0	-15.8	-16.9	-1.2	-2.6	-3.5	-6.8	-16.0	-9.1	-16.9	-15.9	-16.5	-10.8	-18.0	-16.2
February	-16.3	-11.3	-14.7	-14.5	-4.0	-1.5	2.3	-4.6	-15.0	-10.0	-14.3	-13.8	-16.3	-10.2	-15.0	-15.6
March	-7.1	-4.4	-4.7	-6.2	5.4	6.2	4.9	4.6	-5.3	-1.6	-3.4	-5.1	-7.2	-3.7	-3.3	-6.4
April	7.4	10.7	9.0	5.5	12.8	15.7	15.4	12.5	10.1	11.0	9.9	7.1	7.4	10.1	9.7	6.0
May	11.2	17.5	16.7	13.6	17.6	20.8	18.1	17.2	10.4	16.1	17.7	15.4	10.7	15.3	16.4	13.6
June	22.6	20.5	23.0	18.8	24.5	25.6	25.6	22.1	22.8	21.6	21.0	21.0	21.1	20.3	22.0	18.7
July	21.7	20.7	23.6	20.0	25.0	27.7	26.2	24.9	22.4	21.4	25.2	22.3	20.0	19.3	23.4	20.1
Season**	3.1	6.3	3.7	1.4	10.6	10.7	11.2	9.0	3.2	5.0	3.6	3.0	2.1	3.9	3.3	1.6
**Precipitation, mm**																
December	29	19	19	30	65	45	52	41	60	10	49	23	44	17	20	26
January	33	41	13	20	39	34	38	36	45	21	38	21	32	22.1	9	21
February	26	6	37	18	44	17	73	44	20	22	73	21	9	15.8	35	19
March	13	19	25	22	136	77	51	81	18	2	51	22	13	25.9	10	22
April	11	2	16	21	111	57	107	107	15	47	107	25	12	10.7	15	30
May	77	8	16	26	121	80	153	95	49	93	153	36	62	50.8	5	32
June	62	27	22	36	20	17	33	53	65	50	104	42	50	118.0	50	51
July	63	12	78	55	85	10	43	40	126	23	36	64	113	74.0	46.2	76
Season	360	161	302	284	819	481	550	627	549	327	376	331	493	393	275	372

* - long-term average for 2005-24; ** - season from September till July.

Table S12. Traits adjustment in unreplicated trials using check-plot correction.

Institution	Year	Traits adjusted
Kaz. RPC Grain	2024	Winter survival, days to heading, plant height, spike length, spikelets per spike, grains per spike, grain weight per spike, TKW
	2025	-
	2026	Replicated trial – no adjustments
Kaz. RIAPG	2024	Plant height,
	2025	Winter survival
	2026	Days to heading, plant height, spike length, grains per spike, grain weight per spike, grain yield per 1 m^2
Karabalyk AES	2024	Grains per spike, grain weight per spike, TKW
	2025	-
	2026	Replicated trial – no adjustments
North Kaz. AES	2024	Days to heading
	2026	Only 47 entries survived winter – no adjustment.

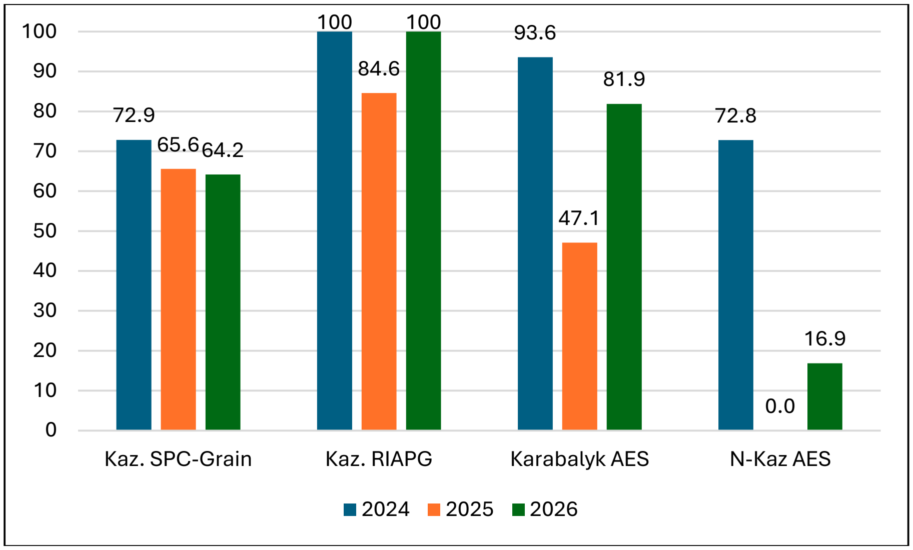

Figure S1. Average winter survival of the germplasm across four sites in 2024-26.

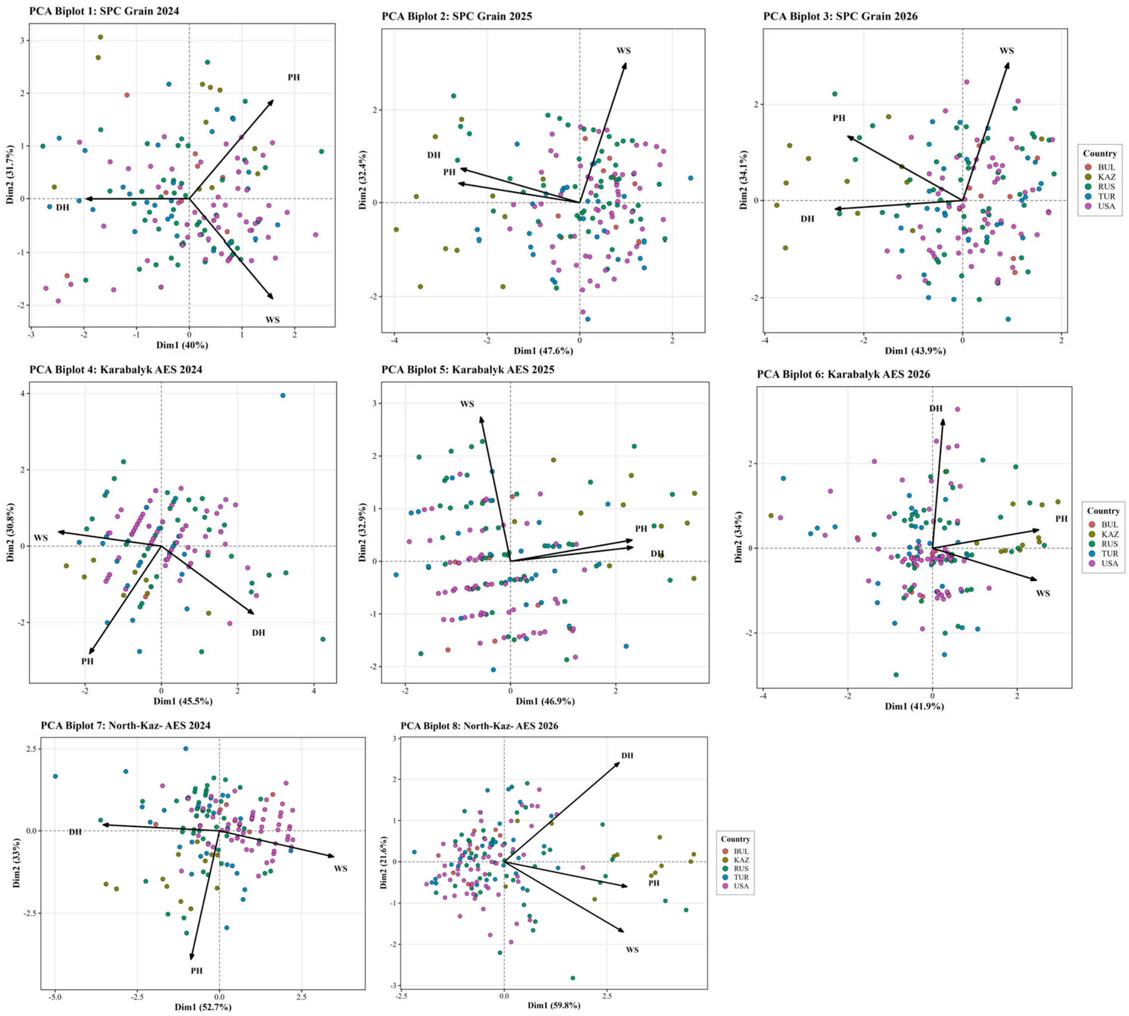

Figure S2. Biplot of winter survival, number of days to heading and plant height at eight trials across three Northern sites in 2024-2026.

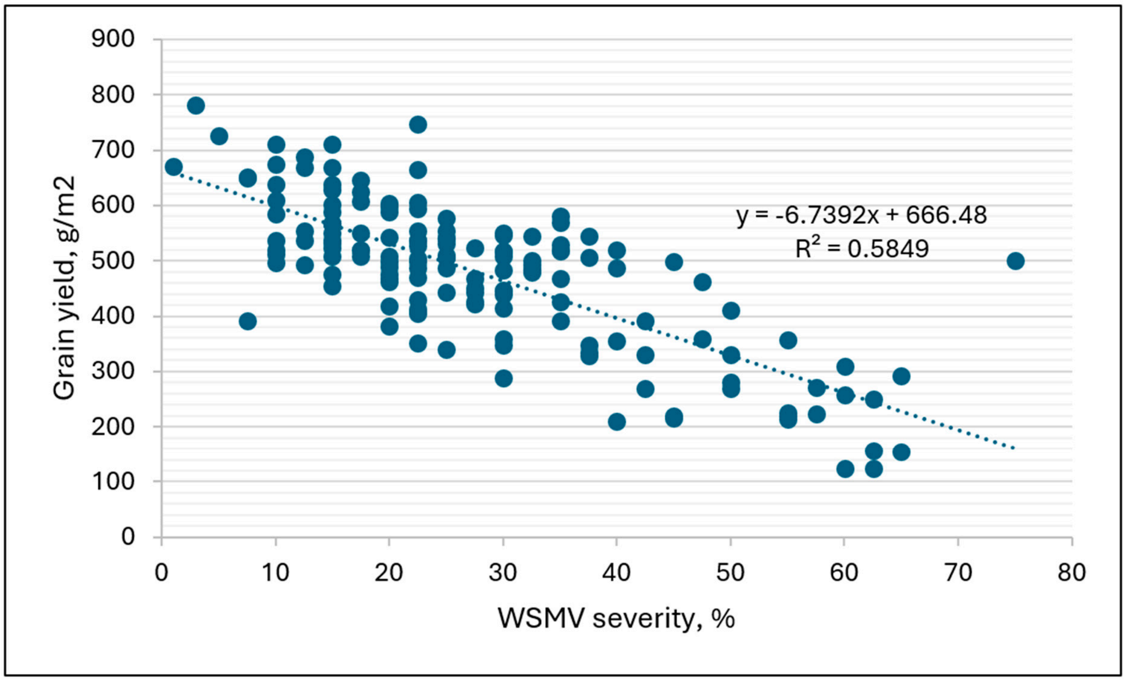

Figure S3. Relationship between WSMV severity and grain yield at Kaz. RPC Grain, 2026.
